# Facilitators of and barriers to interprofessional learning between nurses and physiotherapists in hospital-based clinical practice: A scoping review

**DOI:** 10.1371/journal.pone.0355722

**Published:** 2026-08-07

**Authors:** Kristina Åhlund, Anette Johnsson, Maria Rönnerhag

**Affiliations:** 1 Department of Health Sciences, University West, Trollhättan, Sweden; 2 Department of Research and Development, NU Hospital Group, Trollhättan, Sweden; 3 Department of Orthopaedics, Institute of Clinical Science, Sahlgrenska Academy, University of Gothenburg, Gothenburg, Sweden; Al Azhar University Gaza, PALESTINE, STATE OF

## Abstract

**Introduction:**

Effective collaboration depends on mutual understanding of professional roles and responsibilities, making sustainable interprofessional learning (IPL) an integral part of hospital clinical practice. In this context, learning and collaboration reinforce one another and ultimately support better patient outcomes. However, the factors influencing IPL in hospital settings are not yet clearly understood. Therefore, this scoping review aimed to identify facilitators of, and barriers to, IPL in hospital-based clinical practice, focusing on nurses and physiotherapists.

**Method:**

This scoping review followed the Arksey and O’Malley framework. A systematic literature search was conducted in three databases: PubMed, CINAHL, and Scopus, from inception to July 2026. Inclusion criteria were empirical scientific articles focusing on facilitators and barriers to IPL, applicable to adult hospital settings and including both nursing and physiotherapy perspectives. Grey literature, studies evaluating specific educational interventions or conducted in clinical settings with dedicated IPL initiatives beyond routine daily practice, and studies conducted in paediatric, maternity, or psychiatric settings were excluded. The database search yielded 1,973 articles, of which 11 were included following screening of 33 full-text articles.

**Results:**

The included studies identified a range of facilitators to IPL including factors that supported socialization and familiarization (n = 6), building a positive team climate (n = 5), and conducive organizational structures (n = 10). Barriers included limiting structural prerequisites (n = 5), constraining interprofessional interactions (n = 4), and non-conducive prevailing workplace culture (n = 5).

**Conclusion:**

Sustainable IPL requires organizational structures that enable regular interaction among professionals, inclusive team cultures, and collaborative leadership. This ensures that learning continues even as staff members change, and that essential knowledge is retained in hospital-based clinical practice, so that patients receive care based on their needs. This review may therefore support hospitals and educational organizations in creating conditions that support the systematic integration of IPL into both education and clinical practice and thereby sustain IPL and collaboration between nurses and physiotherapists.

## Introduction

To provide person-centered care for patients with complex needs, the competence of various healthcare professions is essential. Effective interprofessional collaboration (IPC), including shared learning, is considered to reduce healthcare fragmentation and create opportunities to optimize resources, as team members together may achieve more than is possible individually [1,2]. Furthermore, such collaboration is positively associated with patient safety [3]. Achieving effective IPC, however, requires that professionals not only possess discipline-specific expertise but also understand one another’s roles and responsibilities and coordinate their efforts across professional boundaries [4].

In general hospital wards, nurses typically play a key role in coordinating patient care by ensuring that the appropriate professionals are involved [5]. Although medical and nursing staff usually work closely together within the ward, allied health professionals such as physiotherapists are often organizationally affiliated with hospital-wide units and are engaged following referral or contact from ward staff [6]. Within collaborative practice, interprofessional learning (IPL) is actively promoted and recognized as a key success factor for effective, appropriate, safe, and sustainable healthcare [7]. IPL entails professionals from diverse disciplines learning with, from, and about one another to enhance collaboration and mutual understanding [1].

Although the importance of IPL is widely recognized, opportunities for such learning vary across educational and clinical contexts. Traditionally, the education of healthcare professionals has been divided into silos, with one’s own professional role being the focus and the traditions and working methods adopted being based on one’s own tasks [8]. IPC and teamwork are now commonly embedded in the courses of most healthcare education programs, but the forms and arrangements for how the learning takes place vary [9]. Despite increased awareness of IPC, clinical placements often provide medical and nursing students with limited opportunities for structured learning with other professions, including physiotherapists [10]. Furthermore, few referring providers report having received education about physical therapy during their academic or postgraduate training. Notably, those interacting more frequently with physiotherapists perceived greater patient benefit from physiotherapy [11]. These observations highlight the potential importance of learning within clinical practice.

From a sociocultural perspective, learning develops through social interaction and collaboration [12]. Wenger’s theory of communities of practice [13] similarly highlights the role of participation in shared practices and experiences for learning and professional development. In hospital settings, clinical practice provides opportunities for IPL, as healthcare professionals and students develop shared understandings of roles, responsibilities, and patient care through collaborative work [14]. However, such opportunities depend on effective IPC. Research suggests that improving IPC requires coordinated efforts at the organizational, team, and individual levels. Without such combined efforts, opportunities for IPC and, consequently, IPL may be limited [15].

To achieve effective use of healthcare resources and ensure that inpatients at risk of functional decline receive timely physiotherapy assessment and treatment, continuous opportunities for IPL between nurses and physiotherapists is essential. In hospital settings, the daily clinical practice often serves as the primary context for such learning. However, there appears to be limited research on the conditions that facilitate IPL specifically between nurses and physiotherapists. To better understand how IPL unfolds in these clinical settings, a scoping review is therefore warranted to identify factors that facilitate or hinder workplace learning. Such knowledge may guide the development of clinical environments and work practices that support IPL and IPC. This study therefore aimed to enhance our knowledge of IPL in hospital-based practice by identifying facilitators of and barriers to workplace learning, with a specific focus on nurses and physiotherapists. The research questions are accordingly: What are the facilitators of IPL in clinical practice? What are the barriers to IPL in clinical practice?

## Method

The reporting of this scoping review adhered to the Preferred Reporting Items for Systematic Reviews and Meta-Analyses extension for Scoping Reviews (PRISMA-ScR) guideline [16], (S1 File) and the PRISMA 2020 flow diagram for new systematic reviews including searches of databases and registers only [17]. The study protocol was prospectively registered with the Open Science Framework (OSF) (https://doi.org/10.17605/OSF.IO/J52BA). As this study was based exclusively on previously published literature and did not involve human participants, animal subjects, or primary data collection, ethical approval was not required.

To limit subjectivity, the review process was conducted according to the methodological framework of Arksey and O’Malley [18], comprising five major steps: 1) identifying the research questions; 2) identifying relevant studies; 3) selecting the studies; 4) charting the data; and 5) collating, summarizing, and reporting the results. This framework enables the inclusion of empirical studies conducted using different methods, which are identified and analyzed in relation to the research questions.

### Identifying the research question

To guide the development of the research questions and eligibility criteria, the Population–Concept–Context (PCC) mnemonic was applied. The population comprised nurses and physiotherapists, the concept focused on facilitators of and barriers to IPL, and the context was adult hospital settings, defined as care provided to inpatients aged 18 years and older.

### Identifying relevant studies

A systematic literature search was performed in three electronic databases: PubMed (including MEDLINE and PubMed Central), CINAHL, and Scopus, which are considered to encompass interdisciplinary research in health and care involving nursing, rehabilitation, and learning. The search strategy followed the accepted three-step method of a systematic search [19]. An initial limited search was first conducted in PubMed to identify relevant terms and current synonyms. The search string was then developed with support from a librarian, and preliminary searches were carried out in November 2024. The structured search, including all identified keywords and index terms, was subsequently conducted in May 2025, and the search string was then optimized for use in all included databases. The search was subsequently updated in July 2026, yielding no additional studies. Detailed search strategies for all databases are provided in Supporting information (S2 File). Finally, the reference lists of all identified articles were screened for further relevant studies.

The systematic search used the following Boolean search string: (hospital[mesh] OR clinical workplace* OR clinical practice) AND (interprofessional learning OR IPL OR interdisciplinary learning OR interprofessional practice OR multiprofessional practice OR interprofessional collaboration OR multiprofessional collaboration OR multiprofessional team* OR interprofessional collaborative practice) AND (physical therapist* OR physiotherapist* OR paramedic* OR rehab* OR allied*) AND nurs*

### Selecting the studies

Inclusion criteria were peer-reviewed empirical studies published in English from database inception to July 2026, focusing on facilitators of, and/or barriers to IPL in adult hospital settings and including both nursing and physiotherapy perspectives. Grey literature, studies evaluating specific educational interventions or conducted in clinical settings with dedicated IPL initiatives beyond routine daily practice, and studies conducted in paediatric, maternity, or psychiatric settings were excluded. In line with scoping review methodology, no formal critical appraisal of the included studies was undertaken. To ensure a minimum level of scientific quality, eligibility was restricted to peer-reviewed empirical articles.

All identified articles were exported to the Rayyan Screening Tool [20]. Duplicates were excluded during the initial stage of the screening process, and the remaining articles were independently and carefully reviewed by two authors [KÅ, AJ] at the combined title and abstract level, based on the predefined inclusion and exclusion criteria and on the relevance to the study aim. In the next step, full texts were independently reviewed by the same authors, and any disagreements were resolved through discussion, with involvement of the third author [MR], when necessary, until consensus was reached. All co-authors subsequently reviewed the included studies to confirm the accuracy of the selection.

**Fig 1:**
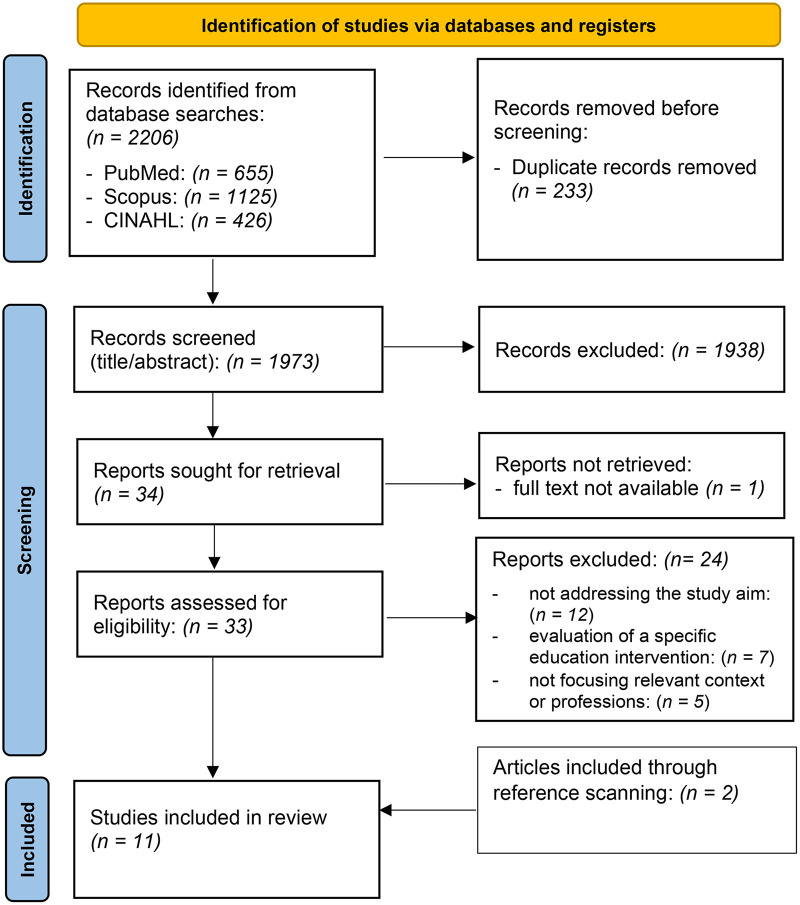
Flowchart.

The database searches identified 2206 articles. After removing duplicates, 1973 titles and abstracts were screened, resulting in 33 articles selected for full-text review. All full texts were carefully read and analyzed based on the study’s aim and the inclusion and exclusion criteria. After screening the reference lists, two additional articles were included. In total, 11 articles were included in the final analysis [21–31]. A PRISMA flowchart [17] summarizing the study selection process is presented in Fig 1.

### Charting the data

A data extraction form was developed using tables in Microsoft Word. The data extraction was carried out collaboratively by the authors: the first author [KÅ] extracted data from all included studies, while the other two authors [AJ and MR] verified the accuracy of the extraction. Data were collected on study characteristics (i.e., author, year, country, study design, context, and participants), and on findings that addressed the study’s research questions regarding facilitators of and barriers to IPL in hospital-based clinical practice, see Table 1.

**Table 1:** Characteristics of the included studies.

Authors, year Title Country	Study design and context	Participants	Facilitators of IPL	Barriers to IPL
**Arndt et al., 2009** [21] *Socialization in health education: encouraging an integrated interprofessional socialization process* Canada	A qualitative interview study with healthcare professionals from urban and rural hospitals and day centers	Thirty-two individual interviews and 14 group interviews (*n* total = 83), representing different clinical healthcare professionals (including nurses and physiotherapists [PTs]) and students	• Familiarization occurs in the placement setting and articulates the differences among professions and establishes an understanding of differences between one another’s professional roles. Familiarization occurs through shadowing and interactions with professionals from different disciplines. • Placement educators play a key role in fostering students’ understanding and ability to work interprofessionally. • Respondents indicated that students primarily learn from and are socialized by discipline-specific placement educators.	• Socialization and familiarization opportunities are not consistently embedded in the curriculum, resulting in varying levels of interprofessional exposure depending on the discipline, setting, and educator. • In placement settings students are unable to build interprofessional skills because of limited opportunities to interact with and learn from other professionals.
**Gum et al., 2012** [22] *From the nurses’ station to the health team hub: How can design promote interprofessional collaboration?* Australia	An ethnographic study including field-work observations and semi-structured interviews with healthcare professionals in rural hospitals	Individual interviews with 33 different clinical healthcare professionals (including nurses and PTs)	• The physical structure of the hospital’s nurses’ station influenced collaborative practice. It was related to the way in which conversations could take place as well as the enablers of and barriers to effective communication. • A PT who provided care in different hospitals explained that she preferred the H1 layout so that she could “yell out to the nurses at the station because they don’t have the glass covers and it is a bit more open to people.”	• The physical structure of the nurses’ stations sometimes negatively influenced the possibility of communicating effectively. • When there was a lack of space in the nurses’ station, many conversations were held in the doorways or corridors or in front of the nurses’ desk, which also affected privacy.
**Hallin & Kiessling, 2016** [23] *A safe place with space for learning: Experiences from an interprofessional training ward* Sweden	A qualitative study based on retrospective analysis of free-text answers in a questionnaire Interprofessional training ward at a university hospital	In total, 333 students in different healthcare education programs (including nurses and PTs)	• Students described the importance of complete student teams in order to learn from one another’s professions. • Students appreciated scheduled opportunities to collaborate, such as structured morning rounds when interprofessional collaboration was emphasized and enough time was allocated. • Communicating and collaborating with other professions provided students with a mirror in which to see themselves in relation to others.	• Lack of students from some professions negatively affected interprofessional collaboration and learning.
**Johannessen & Steihaug, 2014** [24] *The significance of professional roles in collaboration on patients’ transitions from hospital to home via an intermediate unit* Norway	A qualitative study based on interviews with clinical healthcare professionals and observations at an intermediate geriatric unit for patients >60 years with somatic diseases	In total, 38 clinical healthcare professionals (including nurses and PTs)	• The weekly multidisciplinary meeting served as a forum for interprofessional discussions of the unit’s patients.	• The PTs reported that they mainly worked individually, seldom participating in the morning care routine with the nurses. • PTs described collaboration with nurses as important, but sometimes challenging. According to the PTs, there was an “us versus them” attitude in the unit; they found themselves to be excluded from the nurses’ community of practice and felt that they were regarded as guests in the unit. • Observation at the interprofessional meetings showed that perspectives on medical treatment dominated, while issues concerning functional level and recovery were rarely addressed.
**Kvilhaugsvik & Rehnsfeldt, 2019** [25] *Interprofessional learning for nursing students in practice periods: Problems to overcome and possibilities for uniprofessional campuses* Norway	A qualitative study based on focus group interviews with clinical healthcare professionals and teachers in a rural uniprofessional learning environment	Three focus groups, in total 18 participants, involving clinical healthcare professionals from the hospital rehabilitation team (including nurses and PTs)	• IPL in existing teamwork is better than IPL with other students. • In departments where teamwork is less implemented, teachers could ask students to shadow another profession, with a focus on the other profession’s role and responsibilities. • The students’ supervisors introduced students to the teams and included them in the teamwork. • Nursing teachers and supervisors in practice departments have significant influence on whether or not students learn interprofessional collaboration. • Good communication skills and a”we feeling” enable individuals to overcome obstacles and facilitate collaboration. • Communication skills are critically important for IPL. Since the professions originate from different paradigms, equality in dialogue can be challenging.	• Traditional departmental cultures do not always stimulate team competencies and often there is little focus on IPL during student practice periods, despite government requirements. • Most professionals are educated in siloed institutions and are not used to collaborating to the extent suggested by current national guidelines. • Cultures, attitudes, or motivation can govern whether or not teamwork is established.
**Källen et al., 2022** [26] *Content and structure of ward rounds focusing interprofessional collaboration on an internal medicine ward: An observational study of interprofessional collaboration* Sweden	A qualitative study based on observations of ward rounds at an internal medicine ward in a university hospital	Observations of seven healthcare team rounds including 8–12 participants in each (involving nurses and PTs)	• Collaboration required relationships among the team members. • The possibility of digital participation in ward rounds may increase involvement from a broader range of professional groups. • Leadership skills grounded in dialogue are promoted, for example, by asking questions rather than providing answers. • The presence and availability of healthcare professionals enabled joint planning by utilizing their diverse knowledge and skills. • The ward rounds were structured around patients’ self-care and simultaneously functioned as a learning environment where educational perspectives were discussed among team members. This promoted joint planning and care activities by enhancing healthcare professionals’ competence in supporting patients and managing new or unfamiliar treatments.	• Professional hierarchies with main discussions concerning medical conditions led to lack of time to discuss other needs and to passivity among team members. • Rehabilitation and nursing tasks were viewed as more demanding to manage during interprofessional collaboration and the allotted time was not always enough. • Unplanned breaks, such as telephone calls, as well as the shortage of healthcare professionals affected the participating healthcare professionals’ attention during the ward rounds.
**Nisbet et al., 2015** [27] *Interprofessional team meetings: Opportunities for informal* *interprofessional learning* Australia	A qualitative study based on interviews with clinical healthcare professionals and observations of interprofessional team meetings in a tertiary-referral teaching hospital	In total, 77 participants from seven different interprofessional care teams (including nurses and PTs)	• Learning occurred through actively participating and interacting with others: by observing others, listening, and asking questions directly related to patient care, and by participating in interprofessional team discussions. • Attending interprofessional meetings enabled learning about interprofessional communication, which was perceived as different from communicating within one’s own profession due to differences in jargon, terminology, and the need to be succinct. • Face-to-face contact increased familiarity and positively influenced referrals, communication, and confidence. • If leadership encouraged input from all, participation was felt to be positively influenced.	• Individual team members’ level of confidence and experience influenced their capacity to participate in the meeting. There was a tendency to undervalue one’s own knowledge and, as a result, to refrain from contributing to the meeting. • The meeting clearly defined issues from a medical point of view but did not venture outside that clinical viewpoint. This limited participants’ learning about other things, such as discharge planning.
**Rees et al., 2018** [28] *Understanding students’ and clinicians’ experiences of informal interprofessional workplace learning: An Australian qualitative study* Australia	A qualitative interview study conducting both focus group and individual interviews Hospital setting	Twelve group and 10 individual interviews were conducted; healthcare professionals (*n* = 23) and students (*n* = 38) were divided into separate groups (nurses and PTs included)	• Interprofessional student–clinician interactions facilitated student learning and interprofessional attitudes. • Positive contributory factors included motivation, self-awareness, welcoming attitudes and physical space, shared break rooms, and enough time for education. • Learning occurred through, for example, observing clinicians, joint assessments of patients, and discharge planning.	• Negative prior experiences of interprofessional student clinician interactions and lack of time had a negative effect on learning.
**Sinclair et al., 2009** [29] W*hat’s so great about rehabilitation teams? An* *ethnographic study of interprofessional collaboration in a* *rehabilitation unit* Canada	An ethnographic study involving field observations and individual interviews with healthcare professionals from a hospital rehabilitation team	In total, 40 hours of field observations of daily clinical work and 19 individual interviews with clinical healthcare professionals (including nurses and PTs)	• Jointly working with patients encouraged a casual and collegial atmosphere in which team members appeared to be socially and professionally comfortable with one another. • Most team members consistently attended weekly meetings, which seemed to foster a sense of ongoing dialogue and group identity through inculcating shared norms. • Therapists’ need to visit the unit regularly to write in patient charts kept in the nursing station facilitated informal interprofessional exchanges.	• Hierarchies were evident in practice. The team can be described as semi-hierarchical in that no single member of the team was designated as the leader, although some members of the team, because of seniority or clinical role, were treated as having more decision-making authority than others. • No regular meetings were held to exchange information; instead, they were held on demand as needed. • The patient beds were located on the unit, while the therapy rooms, meeting spaces, and most clinician offices were located off the unit. This separation appeared to exacerbate distinctions between professionals and made collaborative decision making difficult.
**Walker et al., 2019** [30] *Students’ experiences and perceptions of interprofessional education during* *rural placement: A mixed methods study* Australia	A mixed-method study comprising a questionnaire including open-ended questions and focus group interviews with students in rural clinical placements	In total, 60 students completed the survey and 11 students participated in three focus group interviews (including nurses and PTs)	• Socializing with students from other professions was valuable for breaking down silos and developing an interprofessional understanding. • Case studies, simulation, shadowing, and joining in interprofessional team meetings were considered good learning activities. • Clinical educators encouraged students to engage with other health professionals.	• Limited opportunities to debrief in interprofessional groups hindered IPL, although it was considered a valuable tool for developing an understanding of others’ professional roles.
**Warshawski, 2016** [31] *The state of collaborative work with nurses in Israel: A mixed method study* Israel	A mixed-method study comprising questionnaires, individual interviews, and observations in a large medical center	Questionnaires completed by 262 professionals, 12 interviews, and 12 observation occasions (1 h each) in hospital wards (nurses and PTs included)		• PTs said that their minimal presence on wards decreased the opportunities for professional and social interactions with nurses; nurses added that the PTs were outsiders and not part of the team. • Nurses’ short staffing and overwork acted as factors undermining collaborative work in the team, especially with nurses. • PTs and nurses worked side by side, sharing and transferring information for common goals, but professional boundaries were maintained.

### Collating, summarizing and reporting the results

The extracted data were tabulated to provide an overview of the included studies and facilitated comparisons of the findings. Guided by the review questions, the data were analyzed and organized according to similarities in content, jointly by all three authors, resulting in themes that facilitated or hindered IPL. The themes were identified and refined before being reported in relation to the aim and research questions of the review.

## Results

In accordance with the methodology used [18], the characteristics of the included studies and the review findings are presented in narrative form. To enhance transparency, detailed data charted from each included study are presented in Table 1, while Table 2 provides an overview of the findings identified across the included studies.

**Table 2:** Overview of facilitators of, and barriers to IPL in clinical practice.

Facilitators of IPL in clinical practice	IPL facilitator content	References
**Supporting socialization and familiarization**	• Interprofessional interactions • Clinical collaboration	[21,23,25,27,28,30]
**Building a positive team climate**	• Meaningful relationships • Well-functioning communication	[25–27,29,30]
**Conducive organizational conditions**	• Encouraging leadership • Shared physical environment • Regular encounters • Digital team meetings	[21–30]
**Barriers to IPL in clinical practice**	**IPL barrier content**	**References**
**Limiting structural prerequisites for learning**	• IPL not clearly formulated in the curricula • IPL based on informal learning • Siloed education	[21,25,28–30]
**Constraining interprofessional interactions**	• Physical separation • Low presence as a barrier to interprofessional interaction	[22,23,29,31]
**Non-conducive prevailing workplace culture**	• A negative ward culture • Team culture	[24,26,27,29,31]

### Study characteristics

In terms of their geographical distribution, studies conducted in Australia were the most common (*n* = 4/11, 36%) [22,27,28,30], followed by Sweden [23,26], Norway [24,25], and Canada [21,29], each represented by two studies, and Israel, represented by one study [31]. All studies were published between 2009 and 2022. The studies employed various methodologies, either individually or in combination. A qualitative design involving individual or focus group interviews was most common (10 out of 11, 91%), followed by field observations (five out of 11, 46%) and surveys (three out of 11, 27%). For the study characteristics, see Table 1.

The sample size ranged from 18 to 333, with study populations representing both genders and diverse backgrounds to illuminate the perspectives of clinicians and students. All included articles studied IPL within teamwork involving both nurses and physiotherapists, among other professions. None of the studies specifically examined learning between these two professions, which is the focus of this review. The included studies encompassed a variety of hospital settings and considered both clinicians’ and students’ experiences of workplace learning in clinical placements. For the study characteristics, see Table 1.

### Facilitators of IPL in clinical practice

The analyzed articles provided insights into the factors facilitating IPL in teams involving nurses and physiotherapists in hospital-based clinical practice. These factors can be summarized into three themes, as described below and in Table 2.

#### Supporting socialization and familiarization.

To promote the exploration of professional roles, clinical interactions and collaboration were described as valuable tools for enhancing mutual knowledge and understanding, enabling socialization and familiarization processes [21,25]. These approaches helped individuals explore their own professional roles by offering a mirror for self-reflection and clarity, while also providing insights into the skills and responsibilities of other professions [21,23,25,27,28,30].

Joint assessments and planning [26,28], shadowing [21,30], observations [27,28], and team rounds [26] were used to enable socialization and familiarization, thereby contributing to increased learning about both one’s own and the others’ professional roles. Being involved in collaborations with others contributed to insight into other professions’ skills and responsibilities [21,26,27] and developed an ability to understand different professional discourses (e.g., terminology) [25,27].

#### Building a positive team climate.

To build a positive team climate, meaningful relationships were found to be important, enhancing confidence within the team and stimulating collaboration, which served as a basis for IPL [25–27,29]. Joint work with patients and regular team meetings encouraged a collegial atmosphere in which team members appeared to be both socially and professionally comfortable with one another [29]. Informal interprofessional exchanges also helped break down silos, for example, when team members got to know one another better, became more confident, and formed a group identity, a sense of “we” [25,29,30].

To create equality in dialogue, it was considered important to develop well-functioning communication as well as confidence in one’s own capacity to actively participate in team meetings, both to learn and to help enhance others’ learning [25]. Being curious and asking questions rather than simply providing answers was also considered important [26].

#### Conducive organizational structures.

The leaders have a major role in encouraging IPL through managing adequate staffing and providing opportunities for regular joint planning that takes different professional needs and perspectives into account [24,27,29]. For students, it is also important to have a clinical supervisor who encourages participation in different interprofessional activities and therefore contributes to students’ IPL during clinical placements [21,25,30].

Shared physical environment, including the design and structure of meeting areas, appeared to be important for creating opportunities to communicate effectively and learn interprofessionally [22]. Being physically co-located, for example, sharing break rooms, stimulated interactions and facilitated communication and collaboration with other professions, enhancing mutual professional understanding [28].

The choice of working methods also influenced IPL in various ways. Regular informal encounters led to scheduled opportunities to collaborate when sufficient time was allocated [23,24]. When therapists came to the ward regularly (e.g., to attend meetings), some informal interprofessional exchanges occurred and members of different professions got to know one another [29]. Videoconference-based team meetings increased accessibility and enabled participation from professionals located off site who otherwise would have been unable to attend [26]. However, face-to-face meetings were perceived as important for strengthening confidence in interactions with other professions, thereby facilitating further contact when needed [27].

### Barriers to IPL in clinical practice

The analyzed articles also identified a number of hindering factors that can be perceived as barriers to IPL in teams involving nurses and physiotherapists. The barriers are summarized in three themes, as described below and in Table 2.

#### Limiting structural learning prerequisites.

When IPL was not explicitly integrated into the curricula, interprofessional understanding was instead developed through informal learning and shaped by the prevailing learning conditions on the ward [21]. Many of the clinicians were previously educated within professional silos and therefore lacked personal experience of structured IPL activities; moreover, negative previous experiences of IPC might negatively affect both professionals’ interest in and ability to organize and attend appropriate IPL activities [25,28].

In the absence of regular interprofessional meetings, clinicians tended to organize meetings when they were perceived as necessary, a process that could easily be challenged by hierarchical structures and lack of time [29]. Furthermore, IPL was diminished when opportunities for structured debriefing within interprofessional groups were limited [30].

#### Constraining interprofessional interactions.

Physical separation could be perceived as a barrier to IPL as it made collaboration and interactions more difficult. Examples of such barriers were situations in which patient beds, therapy rooms, meeting spaces, and clinicians’ offices were not shared areas, thereby exacerbating the distinctions between professions [29]. The design of areas where interprofessional communication took place could also negatively affect the ability to communicate effectively, for example, due to lack of space or privacy [22].

Physiotherapists were perceived as having a low-profile presence on the ward, which reduced their opportunities for both professional and social interactions with nurses. Furthermore, the physiotherapists felt that nurses were sometimes difficult to access due to their high workload, which made collaboration even more challenging [31]. In clinical placements, the clinical educators were responsible for organizing activities that facilitated interaction among students from different professions, such as nursing and physiotherapy. This was perceived as particularly challenging on uni-professional campuses where, for example, physiotherapy students were not present, so nursing students did not naturally engage with them [23].

#### Non-conducive prevailing workplace culture.

The ward culture was perceived as important for the learning atmosphere. A culture that did not stimulate or encourage team competencies hindered both clinicians’ and students’ IPL and contributed to an “us versus them” attitude [24]. Physiotherapists often worked individually and came only when nurses called for them, being perceived as excluded from the nurses’ community of practice and sometimes regarded as guests or outsiders within the unit [24,31].

Hierarchical structures were evident and shaped the team culture, in which certain team members, due to their seniority or specific clinical roles, could be granted greater authority in decision-making processes than others [26,29]. Additionally, a medical focus often dominated multiprofessional meetings, while other perspectives, such as functional status and recovery, were not given sufficient time and attention, thereby negatively impacting learning [24,26,27,29].

The atmosphere within the group influenced both collaboration and learning opportunities. Lack of confidence could lead individuals to undervalue their own knowledge and refrain from contributing [27]. Moreover, unplanned interruptions, such as phone calls, temporary staff absences, or work overload, affected team members’ attention and reduced the time available for interaction [26].

## Discussion

This scoping review, which includes 11 studies, highlights that IPL between physiotherapists and nurses is a dynamic and multifaceted process, deeply influenced by contextual, relational, and organizational factors. In fact, enabling interprofessional interactions is a key component of this process. While the potential for IPL in hospital-based clinical practice is substantial, its implementation remains uneven and often dependent on local conditions. These findings are consistent with previous research conducted across hospital, primary care, and community settings [15]. However, the present study extends existing evidence by specifically focusing on workplace IPL between nurses and physiotherapists in hospital settings.

A central facilitator of IPL is enabling professionals to socialize and familiarize themselves with one another, through interprofessional interaction and clinical collaboration. This aligns with previous research highlighting interprofessional socialization as a key process through which healthcare professionals develop collaborative competence, role understanding, and a shared team perspective [32]. These findings are further supported by Vygotsky’s sociocultural theory [12], which views learning as a social process that develops through interaction and collaboration. By discussing patient care, exchanging perspectives, and reflecting on their professional roles, nurses and physiotherapists develop new understandings of both practice and collaboration. This is also consistent with the idea that learning emerges through participation in shared clinical activities [13]. Through working together, professionals build mutual understanding and shared approaches to patient care, making collaboration itself a source of learning.

Another key facilitator is the development of a collective team identity. Building a positive team climate, characterized by trust, openness, and meaningful relationships, encourages nurses and physiotherapists to engage in shared learning and problem-solving. Informal interactions play a vital role in building this sense of belonging. This further supports the view that learning develops through participation in shared practice [13]. As nurses and physiotherapists work together over time, they develop shared understandings and common ways of working that strengthen IPC and create conditions for IPL.

Conducive organizational structures and support are essential for IPL to thrive. Leadership plays a pivotal role in creating organizational conditions that enable collaboration, while physical co-location facilitates spontaneous interactions and relationship-building. Consistent with these findings, Brand et al. [33] argue that face-to-face encounters should not be deprioritized in the pursuit of greater efficiency, as they remain important for building trust and strengthening IPC.

The results also identified several barriers that hinder the realization of IPL. In an ethnographic research project, Hägg-Martinell et al. [10,34] demonstrated that the ward culture and past experiences influenced opportunities to learn. This is consistent with barriers identified in the present review. Consequently, healthcare personnel with older educational backgrounds or limited prior experience of IPL often rely on prevailing collaborative practices in the workplace as their primary frame of reference. This understanding is frequently passed on to new staff and students, meaning that knowledge of IPL is largely informal and shaped by ward culture, attitudes, and observed behaviors.

In the absence of regular interprofessional meetings, effective collaboration may be hindered by limited awareness of other professionals’ roles and expertise. This aligns with Wikman et al. [35], who showed that the absence of regular interprofessional encounters and structured debriefings restricted opportunities for reflection and shared learning. Additionally, hierarchical structures, time constraints, and physical separation between professions further hinder spontaneous collaboration and are perceived as barriers to IPL.

The identified barriers highlight the importance of creating opportunities for dialogue to support mutual understanding and IPC. Moreover, a workplace that does not actively promote team competencies may contribute to an “us versus them” mentality, marginalizing the roles of certain professionals, particularly physiotherapists, who may be perceived as outsiders. Taken together, these findings support previous research emphasizing that IPC requires active engagement from healthcare professionals [36]. Our results suggest that this engagement involves building trust, fostering meaningful interactions, and coordinating contributions to patient care. In the long term, failing to create conditions that support IPL poses risks to patient safety [3], leading to difficulties working toward shared goals whereby patients’ needs are addressed.

### Implications for practice, education and research

Notably, many of the identified facilitators and barriers were organizational in nature, highlighting the importance of the work environment and structural conditions in supporting workplace IPL. Consistent with previous research, the findings suggest that IPL is promoted when organizational conditions enable healthcare professionals to interact, exchange perspectives, and participate in shared clinical activities [15]. Healthcare managers and policymakers therefore play an important role in reducing structural barriers and creating conditions that support both collaboration and learning in clinical practice [37]. In addition, the findings also have implications for education. Given the limited availability of formal IPL activities during clinical placements, educational programs and clinical supervisors may need additional support to facilitate the development of interprofessional competencies [38]. Furthermore, as evidence regarding IPL involving nurses and physiotherapists remains limited, further research is needed to explore how workplace IPL develops in hospital-based clinical practice and how organizational conditions best support such learning.

### Strengths and limitations

To our knowledge this is the first scoping review mapping facilitators of and barriers to IPL in hospital settings with a focus on clinically active nurses, physiotherapists, and associated students. Other strengths of this study relate to the use of a structured methodological framework for conducting scoping reviews [18], and to the search string development being supported by a university librarian. Furthermore, clarity and transparency were ensured by adherence to a pre-registered study protocol.

However, the results must be considered in light of certain limitations. Despite a systematic search and selection process, there was always the risk of missing eligible studies. The restriction to English-language publications and three databases may also have resulted in the exclusion of relevant evidence and thereby influenced the scope of the review. Furthermore, in line with scoping review methodology and the Arksey and O’Malley framework, no formal quality assessment of the included studies was conducted. While this is a recognized feature of scoping reviews, the findings should therefore be interpreted with consideration of the potentially varying methodological quality of the primary studies. Another limitation was that no studies were found that specifically focused on learning between nurses and physiotherapists; however, both professions were represented in all included studies. The opportunities for learning are likely similar for other professional groups working within hospital care, and the facilitators and barriers described in this scoping review likely highlight opportunities and challenges for IPL among professionals operating across organizational boundaries within the hospital.

## Conclusion

IPL in teams involving nurses and physiotherapists is strengthened by organizational structures that support shared learning environments, inclusive team cultures, and leadership that values collaboration. Facilitators include the systematic integration of IPL into education and clinical practice, shared physical spaces that promote interaction, and leadership that encourages equal participation across professions. However, barriers such as silo-based educations, hierarchical team dynamics, physical separation, and lack of time and continuity hinder collaboration and reduce motivation to engage in IPL.

To address these barriers, hospital organizations and educational institutions must create conditions that support both structured and informal learning, reflective practice, and mutual understanding. Sustainable IPL structures embedded in clinical practice and maintained despite staff turnover are essential for preserving and developing interprofessional knowledge. When these elements are in place, IPL contributes to improved teamwork, enhanced communication, and more cohesive, safe, and person-centered care, enabling patients to receive care that meets their complex needs. Without such commitment, the potential of IPL to improve patient outcomes may remain unrealized.

## Supporting information

S1 File The PRISMA-ScR guideline. (PDF)

S2 File Search strategy. (DOCX)
