# Supplementary material for: Facilitators of and barriers to interprofessional learning between nurses and physiotherapists in hospital-based clinical practice: A scoping review
Source: PLoS One. 2026 Aug 7;21(8):e0355722. doi: 10.1371/journal.pone.0355722 (PMC13450769; doi:10.1371/journal.pone.0355722)
Supplement: Supplementary file 2 [file pone.0355722.s002.docx]

**Search Strategies**

**Database:** PubMed

**Platform:** National Library of Medicine

**Date searched:** May 23, 2025 (initial search) and July 6, 2026 (updated search)

**Time span:** Inception to July 2026

**Language** **limits**: English

**Document** **types**: All document types in the initial search

**Records Retrieved:** n = 655 (initial search), the updated search was limited to last year, n= 126

**Search:** (hospital[mesh] OR clinical workplace* OR clinical practice) AND (interprofessional learning OR IPL OR interdisciplinary learning OR interprofessional practice OR multiprofessional practice OR interprofessional collaboration OR multiprofessional collaboration OR multiprofessional team* OR interprofessional collaborative practice) AND (physical therapist* OR physiotherapist* OR paramedic* OR rehab* OR allied*) AND nurs*

**Database:** CINAHL with fulltexts

**Platform:** EBSCO host

**Date searched:** May 23, 2025 (initial search) and July 6, 2026 (updated search)

**Time span:** Inception to July 2026

**Language** **limits**: English

**Document** **types**: Academic journals in the initial search

**Records Retrieved:** n = 426 (initial search), the updated search was limited to last year, research articles, English language and peer-reviewed, n = 57

**Search:** (hospital OR clinical workplace* OR clinical practice) AND (interprofessional learning OR IPL OR interdisciplinary learning OR interprofessional practice OR multiprofessional practice OR interprofessional collaboration OR multiprofessional collaboration OR multiprofessional team* OR interprofessional collaborative practice) AND (physical therapist* OR physiotherapist* OR paramedic* OR rehab* OR allied*) AND nurs*

**Database:** Scopus
**Platform:** Elsevier
**Date searched:** May 23, 2025 (initial search) and July 6, 2026 (updated search)
**Time span:** Inception to July 2026
**Language limits:** English
**Document types:** All document types in the initial search.

**Records retrieved:** n = 1125 (initial search), The updated search was limited to last year, articles and English language, n = 240

**Search:** (hospital OR clinical workplace* OR clinical practice) AND (interprofessional learning OR IPL OR interdisciplinary learning OR interprofessional practice OR multiprofessional practice OR interprofessional collaboration OR multiprofessional collaboration OR multiprofessional team* OR interprofessional collaborative practice) AND (physical therapist* OR physiotherapist* OR paramedic* OR rehab* OR allied*) AND nurs*
