# Supplementary material for: Facilitators of and barriers to interprofessional learning between nurses and physiotherapists in hospital-based clinical practice: A scoping review
Source: PLoS One. 2026 Aug 7;21(8):e0355722. doi: 10.1371/journal.pone.0355722 (PMC13450769; doi:10.1371/journal.pone.0355722)
Supplement: Supplementary file 3 [file pone.0355722.s003.docx]

**Manuscript Review: Facilitators of and barriers to interprofessional learning between nurses and physiotherapist in clinical hospital practice: A scoping review**

**General comment**

1. As this review aims to identify facilitators and barriers to interprofessional learning between nurses and physiotherapists in clinical hospital practice, I recommend structuring the findings accordingly. This structure would improve clarity, readability, and alignment with the review objectives.

2. Please provide reference for the statement provided in the results and discussion.

3. I recommend professional English language editing to enhance the clarity, coherence, and readability of the manuscript.

**Abstract**

- The title is not specific to older adults and could be revised to better reflect the study scope. Consider shortening the Background section of the abstract and including key methodological details, such as the review period and the number of databases searched, to improve clarity and completeness.
- You may consider to include the future direction of your study in the abstract.

**Introduction**

- The Introduction is rather lengthy. Consider condensing the background information and focusing on the key rationale, research gap, and objectives of the review.
- Rather than providing extensive background information, the authors should critically highlight the existing knowledge gap, the fragmented nature of the evidence, and the rationale for undertaking a scoping review.

**Methods**

- Line 99-100: The manuscript follows the Arksey and O’Malley framework; however, the subheadings are not aligned with its methodological stages. Consider revising the subheadings accordingly. Please include the PRISMA checklist in the supplementary.
- Line 105 – 108: You may consider to include the details of PCC mnemonic that used in this study to improve reader’s understanding.
- Line 111 – 113: Please include the reason of database selection and study duration.
- Line 128 – 130: The term “somatic adult hospital context” may not be familiar to an international audience. Consider replacing it with “adult hospital settings involving patients with physical health conditions” for greater clarity. You may consider to include the age range of this population.
- Line 130 – 131: The inclusion criteria are not clearly described and should specify based on the PCC mnemonic. The authors should also clearly state the type of evidence sources (quantitative/ qualitative/ mixed methods studies). I would recommend explicitly defining interprofessional learning (IPL) because some studies may use terms such as *interprofessional education (IPE), workplace-based learning or interprofessional training*.
- Line 131 – 134: The exclusion criteria should also be clearly described including study design, settings, populations, publication.
- Please clarify whether a quality assessment was undertaken. If not, provide a justification, as quality appraisal is not always required in scoping reviews but should be explicitly addressed.

**Results**

- I would suggest that the first results subheading focus solely on the characteristics of the included studies. The findings related to facilitators and barriers should be presented under separate subheadings to improve clarity and organization of the results.
- Study selection: Please specify whether studies from other methods (grey literature) in the search strategy. If excluded, provide a rationale for this decision
- Line 187: Please specify the age range and gender of the participants included in the review.
- Line 237: The term “digital team meetings” requires further clarification. Please describe the format of these meetings such as videoconferencing, virtual case discussions or online interprofessional learning session.

**Discussion**

- Line 360: The authors should consider discussing the potential limitations associated with restricting the search to specific languages and databases. These methodological choices may have excluded relevant evidence and influenced the overall scope and completeness of the review.
