# Supplementary material for: Facilitators of and barriers to interprofessional learning between nurses and physiotherapists in hospital-based clinical practice: A scoping review
Source: PLoS One. 2026 Aug 7;21(8):e0355722. doi: 10.1371/journal.pone.0355722 (PMC13450769; doi:10.1371/journal.pone.0355722)
Supplement: Supplementary file 4 [file pone.0355722.s004.docx]

## **Manuscript Review: Facilitators of and barriers to interprofessional learning between nurses and physiotherapist in clinical hospital practice: A scoping review**

**Dear Editor,**

Thank you for the time and effort invested in reviewing our manuscript and for giving us the opportunity to revise it in response to the reviewers’ constructive comments and suggestions.

We believe that the revision has substantially improved the manuscript by clarifying the scope and rationale of the study, strengthening its methodological transparency and rigor, and improving the overall structure and readability. We have carefully considered all comments and revised the manuscript accordingly.

Please find below our point-by-point responses to the reviewers’ comments, together with a description of the revisions made. For ease of review, our responses to the reviewers’ comments are provided in red. All page and line references refer to the updated version of the manuscript *(Manuscript)*. In the *Revised Manuscript Track Changes* document, deleted text is shown as strikethrough, while all additions and revisions are highlighted in red.

We hope that you and the reviewers will find that the manuscript has been significantly strengthened and is now suitable for publication in PLOS ONE.

Sincerely,

Kristina Åhlund

**General comments**

- Please replace Figure 1 with the appropriate PRISMA diagram from the link: https://www.prisma-statement.org/prisma-2020-flow-diagram

Please find the appropriate PRISMA diagram for your study, download the Word document, and fill it out. Please state in your Methods section that you have followed this guideline and provide a reference citation and cite the completed checklist as a supplementary file.
Thank you for noticing this. We have now updated the Flowchart to the correct form according to PRISMA and added a statement in the Method section that we followed that guideline (see, p.6, lines 102-103 and Fig 1)

- Clearly highlight the inclusion and exclusion criteria of the included studies and the included population.

Thank you for this valuable comment. We agree and have revised the manuscript to provide a clearer description of the inclusion and exclusion criteria. Please see, pp.7-8, lines 139-146.

- Identify what “The population, concept, and context (PCC) mnemonic” is and how it was applied in the study.
  We agree that the description was unclear. We have now revised the manuscript to provide a clearer explanation of how the PCC mnemonic was applied. Please see p.6, lines 114-117.

**Reviewer 1**

**General comments**

1. As this review aims to identify facilitators and barriers to interprofessional learning between nurses and physiotherapists in clinical hospital practice, I recommend structuring the findings accordingly. This structure would improve clarity, readability, and alignment with the review objectives.

Thank you for your valuable suggestions regarding the structure of the findings. In accordance with your comments, we have clarified the structure of the Results section (pp.9-14) to improve the clarity and readability of the manuscript. We have also revised Table 2 accordingly to ensure consistency throughout the manuscript.

2. Please provide reference for the statement provided in the results and discussion.
   Thank you for noting this. We identified that references were missing for a few statements in the Results section. These have now been added, and the manuscript has been revised accordingly (p.11, line 209 and p.12, lines 239 and 241).
3. I recommend professional English language editing to enhance the clarity, coherence, and readability of the manuscript.

Thank you for your attention to this matter. As English is not the authors’ first language, the manuscript underwent professional language editing prior to submission. We have now carefully reviewed the manuscript once more and corrected the linguistic issues identified. We hope that the revised version is now clearer and linguistically improved.

**Abstract**

- The title is not specific to older adults and could be revised to better reflect the study scope.

Thank you for bringing this to our attention. As it was unclear which older population the study focused on, and since our methods did not restrict the population beyond adults (18 years and older), we have removed the specific focus on older adults and instead refer to adult in-hospital care throughout the manuscript. Consequently, we have not revised the title as suggested.

- Consider shortening the Background section of the abstract and including key methodological details, such as the review period and the number of databases searched, to improve clarity and completeness.

Thank you for this suggestion. Following your recommendation, we have shortened the Introduction section of the abstract to clarify the study rationale and expanded the methods section to improve the description of how the study was conducted, see the Abstract

- You may consider to include the future direction of your study in the abstract.
  We agree that the future directions were not sufficiently clear. We have now included a statement in the concluding section of the abstract outlining future directions and the potential implications of the study. Please see the Abstract.

**Introduction**

- The Introduction is rather lengthy. Consider condensing the background information and focusing on the key rationale, research gap, and objectives of the review.

Rather than providing extensive background information, the authors should critically highlight the existing knowledge gap, the fragmented nature of the evidence, and the rationale for undertaking a scoping review.

Thank you for this valuable comment. Upon revisiting the Introduction, we agreed that it was unnecessarily long and did not sufficiently emphasize the knowledge gap and the rationale for conducting a scoping review. The Introduction has therefore been substantially shortened and refocused. Text that did not directly contribute to the study rationale was removed to better highlight the knowledge gap and the need for this review. Please see the revised manuscript (pp. 4–5).

**Methods**

- Line 99-100: The manuscript follows the Arksey and O’Malley framework; however, the subheadings are not aligned with its methodological stages. Consider revising the subheadings accordingly. Please include the PRISMA checklist in the supplementary.

Thank you for drawing our attention to this issue. We have now revised all method section subheadings to align with the Arksey and O’Malley framework. During this revision, we also recognized that Step 5 (Collating, Summarizing, and Reporting the Results) was previously insufficiently described. We therefore expanded this section to provide a clearer account of the analytical process and the reporting of the findings (see the manuscript, pp.6-9). We have also updated and included the PRISMA checklist as Supporting information (S1).

- Line 105 – 108: You may consider to include the details of PCC mnemonic that used in this study to improve reader’s understanding.

Thank you. As mentioned above, we have now revised the manuscript to provide a clearer explanation of how the PCC mnemonic was applied. Please see p.6, lines 114-117.

- Line 111 – 113: Please include the reason of database selection and study duration.

Thank you for this valuable comment. In response, we have clarified the rationale for the selection of databases and the chosen time frame in the Methods section of the manuscript (p. 8, lines 161–172).

- Line 128 – 130: The term “somatic adult hospital context” may not be familiar to an international audience. Consider replacing it with “adult hospital settings involving patients with physical health conditions” for greater clarity. You may consider to include the age range of this population.

Thank you for drawing our attention to this issue. We have now revised the description of the study setting to “adult hospital settings” or hospital-based clinical practice throughout the manuscript, including the abstract.

We considered a detailed explanation of somatic care unnecessary after explicitly adding psychiatric settings to the exclusion criteria (p.8, line 143). Furthermore, we removed the specific focus on older adults because it was not supported by our eligibility criteria, and added the age limit 18 years and older (p.6, line 117)

- Line 130 – 131: The inclusion criteria are not clearly described and should specify based on the PCC mnemonic. The authors should also clearly state the type of evidence sources (quantitative/ qualitative/ mixed methods studies). I would recommend explicitly defining interprofessional learning (IPL) because some studies may use terms such as *interprofessional education (IPE), workplace-based learning or interprofessional training*.

Thank you for this comment. We have revised the eligibility criteria to more explicitly reflect the PCC framework, clarifying the population (nurses and physiotherapists), concept (facilitators and barriers to IPL), and context (adult hospital settings). We have also specified that empirical studies only were eligible for inclusion, regardless of methodological approach. Furthermore, IPL is defined in the Introduction section (p.4, lines 62-64) and is now more clearly reflected in the search strategy. As the review specifically focused on workplace-based IPL rather than educational interventions, IPE-related terms were not included in the search strategy (pp.7-8, line 138-145).

- Line 131 – 134: The exclusion criteria should also be clearly described including study design, settings, populations, publication.

Thank you for this comment. We agree and have revised the exclusion criteria to more clearly specify which studies were excluded (pp.7-8, line 140-145).

- Please clarify whether a quality assessment was undertaken. If not, provide a justification, as quality appraisal is not always required in scoping reviews but should be explicitly addressed.

Thank you for this comment. We acknowledge the importance of study quality. However, in line with current scoping review methodology, a formal quality appraisal of included studies was not undertaken. To support scientific rigor, we restricted the inclusion criteria to peer-reviewed empirical studies. This rationale has now been clarified in the Methods section of the manuscript (p.8, lines 143-145).

**Results**

- I would suggest that the first results subheading focus solely on the characteristics of the included studies. The findings related to facilitators and barriers should be presented under separate subheadings to improve clarity and organization of the results.

Thank you for this comment. The text previously included in the Results section has been moved to the Methods section (Selecting the studies). We agree that the structure of the Results section needed to be clarified. We have therefore reorganized the section under the main headings Characteristics of the Included Studies, Facilitators of IPL in clinical practice, and Barriers to IPL in clinical practice, each with relevant subheadings. We believe this revision improves the clarity and organization of the findings (see, pp9-14).

- Study selection: Please specify whether studies from other methods (grey literature) in the search strategy. If excluded, provide a rationale for this decision

Thank you for this comment. It has now been clarified that grey literature was excluded because it had not undergone peer review, which was considered important for ensuring the quality of the included evidence. This has been specified in the manuscript (p. 8, lines 144–145).

- Line 187: Please specify the age range and gender of the participants included in the review.

Thank you for this observation. As the review included only healthcare professionals, all participants were adults of working age. Since Table 1 already contains extensive information and may be considered relatively dense, we chose not to further emphasize participants’ age and gender characteristics in the individual studies.

- Line 237: The term “digital team meetings” requires further clarification. Please describe the format of these meetings such as videoconferencing, virtual case discussions or online interprofessional learning session.

Thank you for noting this. We have now clarified in the manuscript that the digital team meetings referred to videoconferencing meetings (p.12, line 246)

**Discussion**

- Line 360: The authors should consider discussing the potential limitations associated with restricting the search to specific languages and databases. These methodological choices may have excluded relevant evidence and influenced the overall scope and completeness of the review.

Thank you for highlighting this important issue. The limitations related to the restriction to English-language publications and the selection of databases have now been acknowledged and clarified in the Limitations section of the manuscript (p.18, lines 377-390)

**Reviewer 2**

I would like to commend the authors for choosing a highly relevant and timely topic. This scoping review addresses a critical gap in health services literature by mapping the facilitators of and barriers to interprofessional learning specifically between nurses and physiotherapists in clinical hospital settings. The manuscript exhibits notable strengths, including a solid methodological foundation built on established scoping review frameworks, strict adherence to PRISMA-ScR guidelines, and proactive prospective protocol registration.

The thematic synthesis of organizational conditions, team climates, and structural barriers is logically organized, providing valuable, actionable insights for clinical educators and hospital administrators alike. The manuscript holds clear merit and aligns well with the rigorous scope of PLOS ONE.

Despite these clear strengths, the manuscript requires a few essential revisions before it can be considered suitable for final publication. These recommendations primarily focus on correcting critical journal policy compliance issues, elevating methodological transparency, adding explicit data metrics with illustrative examples in the results, and deepening the overall analytical rigor of the discussion section. To assist the authors in maximizing the scholarly impact and readability of their research, I have outlined the essential revisions below.

1. **Methodology and Data Support**

- Quality Assessment: The manuscript does not address whether the included studies underwent quality appraisal. While quality assessment is not required for scoping reviews, acknowledging this limitation would enhance transparency.

You can add the following statement: "Consistent with scoping review methodology and the Arksey and O'Malley framework, formal quality assessment of the included studies was not performed. While this is a recognized feature of scoping reviews, readers should interpret the findings with consideration of the varying methodological quality of the primary studies."
Thank you for noting this important point. We have revised the manuscript accordingly. Specifically, we clarified how we addressed the quality of the included studies (p.8 lines 143-145). We have also incorporated the proposed text into the Limitations section of the Discussion to acknowledge the absence of a formal quality assessment and its implications for the interpretation of the findings (p.18, lines 380-384).

**Enhance Transparency:**

- Detail the screening consensus process, clarify if inter-rater reliability metrics (e.g., Cohen's kappa) were calculated, and justify omitting formal quality appraisal and statistical testing given the scoping review design.
  Thank you for this comment. We have clarified the screening and consensus process in the Methods section. Title- and abstract screening was conducted independently by two authors based on predefined inclusion and exclusion criteria, followed by full-text assessment. Any uncertainties were discussed with the third author until consensus was reached. Given the clear eligibility criteria and the narrative nature of the scoping review methodology, no inter-rater reliability statistics were calculated (pp. 7-8).
- We have also clarified that inclusion was restricted to peer-reviewed empirical studies. Consistent with scoping review methodology, no formal quality appraisal was conducted. To address the implications of this approach, as mentioned above, we have expanded the Limitations section and discussed how the absence of formal quality assessment may affect interpretation of the findings (p.18, lines 380-384).

**Risk of bias:**

- The review process involved independent screening by two authors, which is commendable. However, the manuscript could benefit from clearer description of:

How disagreements were resolved during title/abstract screening

Whether inter-rater reliability was calculated
Thank you for this comment. These issues raised have been addressed in our response above.

The specific roles of each author in data extraction verification

Thank you for this comment. The data charting process is now clarified under the heading “Charting the data”. Briefly, all data were charted by the first author and subsequently reviewed and verified by the two co-authors to ensure consistency and accuracy in the extracted data (pp. 8-9, lines 163-168).

**Strengthen Evidence and Metrics:**

- Provide concrete examples from primary studies and state the exact study counts ($n$) contributing to each theme in both the Abstract and Results sections.
  Thank you for this suggestion. We have added information to the abstract indicating the number of studies represented by each theme. In the Results section (pp.9-14) and in Table 2, however, we believe that the studies underpinning each part of the findings are already reflected through the references provided in the text.

**Deepen Discussion**:

- The Discussion currently tends to restate findings rather than analyze them in broader context. The connection to Vygotsky and Wenger is appropriate but could be more thoroughly developed. Move beyond descriptive summary by fully integrating Vygotsky’s and Wenger’s frameworks to explain findings, translating them into actionable recommendations for practice, education, and health policy. Briefly compare findings with other scoping reviews or systematic reviews on IPL in other contexts (e.g., primary care, community settings) to highlight what is unique about the hospital clinical practice context.
  Thank you for this suggestion. The discussion has been substantially revised to reduce the repetition of the study findings and to place greater emphasis on their analysis and interpretation in relation to theory and previous evidence. (pp.14-18). Furthermore, a separate section outlining implications for practice, education, and research has been added to provide actionable recommendations for supporting IPL in hospital settings (p.17, lines 356-369).
- The Results section presents findings through aggregated themes (facilitators: 3 themes; barriers: 3 themes) with multiple references per theme. While this is appropriate for scoping reviews, it lacks specific illustrative examples that would help readers understand how these factors manifest in practice.
  Thank you for this valuable comment. We agree that illustrative examples may help readers better understand how facilitators and barriers manifest in practice. However, following the methodological framework proposed by Arksey and O’Malley, the purpose of a scoping review is to provide a descriptive summary and mapping of the evidence. We therefore chose to present the findings as aggregated themes summarizing evidence across the included studies.

However, to ensure transparency, we have chosen to retain the comprehensive Table 1, which provides detailed information on all included studies and the data charted from each of them. We therefore believe that the current presentation is consistent with the aim and methodology of a scoping review while allowing readers to trace the evidence underlying each theme.

- Expand the implications section to provide concrete recommendations:

For hospital administrators: specific strategies for creating shared physical spaces, scheduling regular interprofessional meetings, and leadership training.

For educators: how to better prepare students for interprofessional practice and support clinical supervisors.

For policymakers: implications for national guidelines and accreditation requirements.
Thank you for this very valuable comment. As mentioned above, we have expanded the Discussion section by adding a subsection on “Implications for practice, education and research” (p.17, lines 356-369).

2. **Policy and Data Availability**

- **Correct Data Statement:**
  Revise the Data Availability Statement to reflect that all underlying data are within Tables 1 and 2, removing inaccurate references to non-existent supporting files.

"All relevant data are within the manuscript and its Supporting Information files."

The authors claim data is in "Supporting Information files", but no such files were actually submitted with the manuscript.

It can be replaced by "All data generated or analyzed during this study are included in this published article (Table 1 and Table 2). No additional Supporting Information files are associated with this manuscript."
Thank you for this suggestion. Following valuable feedback received during the review process, we further developed the PRISMA checklist and the search strategy documentation. These materials have now been included as Supporting materials (S1 and S2) to enhance transparency and reproducibility. In addition, a Supporting Information section has been added to the end of the manuscript to ensure compliance with PLOS ONE publication guidelines (p.19, lines 417-419)

- **Submit Missing Visuals:**
  Upload the referenced PRISMA flowchart (Figure 1) as a separate, high-resolution image file instead of using a text placeholder.
  Thank you for this suggestion. We have now updated the flow diagram to a PRISMA flowchart and uploaded it as a separate file.
- **Include Mandated Declarations:**
  Add an explicit Ethics Statement to the Methods section clarifying that ethical approval was not required for this secondary review of published literature.

You can add the following statement: "As this study constitutes a scoping review of previously published literature and did not involve human participants, animal subjects, or primary data collection, ethical approval was not required."

Thank you for this valuable suggestion. The proposed text has now been added to the beginning of the Methods section to further clarify the ethical approach (p.6, lines 104-106).

- **Optimize Documentation:**
  Consider moving full, database-specific search strings and raw extraction quotes into a dedicated Supporting Information file for complete replicability.

Thank you for this suggestion. We have now increased the transparency of the search process by including an overview of the search strategies as supporting information (S2).

3. **Language, Formatting, and Presentation**

- Several language errors and awkward constructions throughout the manuscript. Inconsistent use of "interprofessional" vs. "inter-professional" (with hyphen) throughout the manuscript. Standardize it.
  Thank you for your attention to this matter. As English is not the authors’ first language, the manuscript underwent professional language editing prior to submission. We have now carefully reviewed the manuscript once more and corrected the linguistic issues identified. We hope that the revised version is now clearer and linguistically improved.
- Reformat Tables: Table 1 is dense and may be difficult for readers to navigate due to the amount of text in the "Facilitators" and "Barriers" columns. You can split it to tables.
  Thank you for this suggestion. We agree that the readability of the table is important. However, we also consider it important that the table remains comprehensive and presents key data from the included studies. Therefore, we have chosen to retain the current format.
- Verify Citations: Some references do not fully conform to PLOS ONE's reference formatting requirements.

Ref 1: Missing publisher location details

Ref 5: Incomplete journal information

Ref 6, 10: Inconsistent presentation of journal names

Format all references to strictly comply with PLOS ONE’s Vancouver style, ensuring full journal names, correct volume/page numbers, and missing DOIs.
Thank you for highlighting this issue. We have now carefully reviewed and revised the reference list to ensure that all references conform to PLOS ONE's formatting requirements.

**Comments according to section:**

**Abstract**: The abstract is well-structured and follows the standard format (Background, Method, Results, Conclusion).

Suggestions for improvement:

Background: Consider adding:

- One sentence on the importance of IPL for patient outcomes based on existing evidence.

Thank you for this suggestion. We have now revised the abstract to include a statement highlighting the potential patient benefits of IPL and interprofessional collaboration (lines 19-20).

Method: Add brief mention of:

- The databases searched

Thank you for this comment. The three databases included in the search strategy are already reported in the Abstract.

- Number of studies included (already included in Results)

Thank you for this comment. To improve the structure and reporting of the review process, information regarding the search results has now been moved from results to methods. Please see the revised abstract.

Results: The results section could be slightly more quantitative:

"The database search yielded 1,973 articles, of which 11 were included. Facilitators included factors supporting socialization (n=X studies), positive team climate (n=X), and organizational conditions (n=X). Barriers included limited structural prerequisites (n=X), restricted interactions (n=X), and non-conducive workplace culture (n=X)."

Thank you for this comment. To address this point, we have revised the abstract to specify the number of studies supporting each identified theme. Please see the revised abstract.

Conclusion: Currently strong. Consider adding a sentence on the implications for policy or practice.

Thank you! To further clarify the future implications of the review, we have added the following sentence to the Abstract: “This review may therefore support hospitals and educational organizations in creating conditions that support the systematic integration of IPL into both education and clinical practice and thereby sustain IPL and collaboration between nurses and physiotherapists” (lines 41-44).

**Introduction**: The introduction effectively establishes the rationale for the study, provides relevant background on interprofessional collaboration, and justifies the focus on nurses and physiotherapists.

1.Add a paragraph on the prevalence of hospitalization among older adults and the consequences of functional decline to strengthen the clinical relevance.
Thank you for this comment. Since it was previously established during the review process that the methodology does not justify a specific focus on older adults, we revised the manuscript to describe the study population as adults. Therefore, we consider this comment to have been addressed and no longer applicable to the revised manuscript.

2.Briefly acknowledge the existing evidence on barriers and facilitators to interprofessional collaboration more broadly to show this review builds on existing knowledge.
Thank you for this comment. We have expanded the introduction section by including additional information and relevant references to previous research, thereby strengthening the justification for undertaking this scoping review, see introduction section, pp.4-5.

**Methods**: Strong methodological foundation.

1.Search strategy details: The search string is provided in text, but a table with the full search strategy for each database would be helpful for replicability. Consider adding as Supporting Information.
Thank you for this comment. We agree that a more detailed description of the search strategy is important. Therefore, additional information regarding the search strategy has been added to the Supporting information (S2) to improve transparency and reproducibility.

2.Gray literature: The manuscript does not explain why gray literature was excluded. Adding a brief justification would be helpful.
Thank you for this comment. Given that formal quality assessment is generally not required within scoping review methodology, we limited inclusion to peer-reviewed scientific articles to ensure a satisfactory scientific standard among the included studies. This rationale has now been clarified in the Methods section pp.7-8, lines 140-145).

3.Scoping review vs. systematic review: Briefly justify why a scoping review is appropriate (e.g., to map the available evidence rather than answer a focused clinical question).
Thank you for this comment. This has now been clarified in the rationale for the study (p.5, lines 90-93).

**Results**: Results are presented clearly with appropriate use of tables.

1.Add a brief description of the thematic analysis process in the Methods section (who developed the themes, how consensus was reached).
Thank you for this comment and for highlighting this issue. We acknowledge that the description of the data analysis was insufficient in the previous version of the manuscript. To address this, we have added a section entitled “Collating, summarizing and reporting the results” to provide a clearer account of the analytical process. While the analysis did not constitute a formal thematic analysis, it followed the descriptive approach commonly applied in scoping reviews and was conducted in accordance with the methodological framework proposed by Arksey and O’Malley. This has now been clarified in the Methods section (p.9, lines 172-177).

2.Provide numbers of studies contributing to each theme (quantitative summary) in addition to the references.
Thank you for this suggestion. We believe that the suggested revision improves the clarity of the abstract and have therefore implemented it (Abstract, lines 34-37). The included studies are identified through citations throughout the Results section, and the number of studies contributing to each theme is clearly presented in Table 2.

**Discussion**: Discussion connects findings to theory and practice but could be strengthened.

1.Compare findings with previous reviews: Briefly discuss how this review's findings align with or differ from previous reviews on interprofessional education and collaboration.
Thank you for this comment. This has now been addressed as described in our response to a previous comment. Please see the Discussion section (pp.14-18)

2.Add a subsection on "Implications for Research":

Need for studies specifically examining nurse-physiotherapist interaction

Need for longitudinal studies examining sustainability of IPL

Need for intervention studies testing specific IPL strategies

3.Add a subsection on "Implications for Education":

How can curricula be adapted to better prepare students for IPL?

What role should clinical supervisors play?

How can university-hospital partnerships be strengthened?

4.Add a subsection on "Implications for Practice":

Concrete recommendations for hospital administrators and ward managers

Specific strategies for overcoming identified barriers

Role of technology in facilitating IPL

Thank you for this comment. We have now clarified the future implications in a separate section of the Discussion. This section outlines implications for practice, education, and research, based on the findings of the review (p.17, lines 356-369).

**Conclusion**: Conclusion is appropriate and summarizes key findings.

Suggested Statement: "Hospital organizations and educational institutions must prioritize the development of sustainable IPL structures that transcend individual professionals and persist through staff turnover. Without such commitment, the potential for IPL to improve patient outcomes for older adults will remain unrealized."

Thank you for this suggestion. We have revised the conclusion to emphasize the need for sustainable IPL structures embedded in clinical practice that are maintained despite staff turnover. We also highlight that organizational commitment from both healthcare and educational institutions is essential for realizing the potential of IPL to improve patient outcomes (p.19, lines 399-406)
