# Supplementary material for: Facilitators of and barriers to interprofessional learning between nurses and physiotherapists in hospital-based clinical practice: A scoping review
Source: PLoS One. 2026 Aug 7;21(8):e0355722. doi: 10.1371/journal.pone.0355722 (PMC13450769; doi:10.1371/journal.pone.0355722)
Supplement: Peer review history

# Peer review: Decision Letter 0

**DOI:** 10.1371/journal.pone.0355722.r001

**Authors:** Suad J Ghaben

1 Jul 2026

PONE-D-25-59222

Facilitators of and barriers to interprofessional learning between nurses and physiotherapists in clinical hospital practice: A scoping review

PLOS One

Dear **Dr. Åhlund,**

Thank you for submitting your manuscript to PLOS ONE. After careful consideration, we feel that it has merit but does not fully meet PLOS ONE’s publication criteria as it currently stands. Therefore, we invite you to submit a revised version of the manuscript that addresses the points raised during the review process.

==============================

- Please replace Figure 1 with the appropriate PRISMA diagram from the link:

https://www.prisma-statement.org/prisma-2020-flow-diagram

- Please find the appropriate PRISMA diagram for your study, download the Word document, and fill it out. Please state in your Methods section that you have followed this guideline and provide a reference citation, and cite the completed checklist as a supplementary file.
- Clearly highlight the inclusion and exclusion criteria of the included studies and the included population.
- Identify what “The population, concept, and context (PCC) mnemonic” is and how it was applied in the study.
- Upon re-submission, authors should make sure they address each of the reviewers' and editors’ comments in a clear, point-by-point way; the changes/amendments must be clearly shown/marked in the resubmitted manuscript to facilitate a quick revision.

==============================

Please submit your revised manuscript by Aug 15 2026 11:59PM. If you will need more time than this to complete your revisions, please reply to this message or contact the journal office at plosone@plos.org. When you're ready to submit your revision, log on to https://www.editorialmanager.com/pone/ and select the 'Submissions Needing Revision' folder to locate your manuscript file.

Please include the following items when submitting your revised manuscript:

- A letter that responds to each point raised by the academic editor and reviewer(s). You should upload this letter as a separate file labeled 'Response to Reviewers'.
- A marked-up copy of your manuscript that highlights changes made to the original version. You should upload this as a separate file labeled 'Revised Manuscript with Track Changes'.
- An unmarked version of your revised paper without tracked changes. You should upload this as a separate file labeled 'Manuscript'.

If you would like to make changes to your financial disclosure, please include your updated statement in your cover letter. Guidelines for resubmitting your figure files are available below the reviewer comments at the end of this letter.

If applicable, we recommend that you deposit your laboratory protocols in protocols.io to enhance the reproducibility of your results. Protocols.io assigns your protocol its own identifier (DOI) so that it can be cited independently in the future. For instructions see: https://journals.plos.org/plosone/s/submission-guidelines#loc-laboratory-protocols. Additionally, PLOS ONE offers an option for publishing peer-reviewed Lab Protocol articles, which describe protocols hosted on protocols.io. Read more information on sharing protocols at https://plos.org/protocols?utm_medium=editorial-email&utm_source=authorletters&utm_campaign=protocols.

As the corresponding author, your ORCID iD is verified in the submission system and will appear in the published article. PLOS supports the use of ORCID, and we encourage all coauthors to register for an ORCID iD and use it as well. Please encourage your coauthors to verify their ORCID iD within the submission system before final acceptance, as unverified ORCID iDs will not appear in the published article. *Only*  the individual author can complete the verification step; PLOS staff *cannot*  verify ORCID iDs on behalf of authors.

We look forward to receiving your revised manuscript.

Kind regards,

Suad J Ghaben, PhD

Academic Editor

PLOS One

Journal Requirements:

When submitting your revision, we need you to address these additional requirements.

1. Please ensure that your manuscript meets PLOS ONE's style requirements, including those for file naming. The PLOS ONE style templates can be found at

https://journals.plos.org/plosone/s/file?id=wjVg/PLOSOne_formatting_sample_main_body.pdf and

https://journals.plos.org/plosone/s/file?id=ba62/PLOSOne_formatting_sample_title_authors_affiliations.pdf

2. Please include your tables as part of your main manuscript and remove the individual files. Please note that supplementary tables (should remain/ be uploaded) as separate "supporting information" files.

3. When completing the data availability statement of the submission form, you indicated that you will make your data available on acceptance. We strongly recommend all authors decide on a data sharing plan before acceptance, as the process can be lengthy and hold up publication timelines. Please note that, though access restrictions are acceptable now, your entire data will need to be made freely accessible if your manuscript is accepted for publication. This policy applies to all data except where public deposition would breach compliance with the protocol approved by your research ethics board. If you are unable to adhere to our open data policy, please kindly revise your statement to explain your reasoning and we will seek the editor's input on an exemption. Please be assured that, once you have provided your new statement, the assessment of your exemption will not hold up the peer review process.

4. Please include captions for your Supporting Information files at the end of your manuscript, and update any in-text citations to match accordingly. Please see our Supporting Information guidelines for more information: http://journals.plos.org/plosone/s/supporting-information.

5. If the reviewer comments include a recommendation to cite specific previously published works, please review and evaluate these publications to determine whether they are relevant and should be cited. There is no requirement to cite these works unless the editor has indicated otherwise.

6. Please review your reference list to ensure that it is complete and correct. If you have cited papers that have been retracted, please include the rationale for doing so in the manuscript text, or remove these references and replace them with relevant current references. Any changes to the reference list should be mentioned in the rebuttal letter that accompanies your revised manuscript. If you need to cite a retracted article, indicate the article’s retracted status in the References list and also include a citation and full reference for the retraction notice.

[Note: HTML markup is below. Please do not edit.]

Reviewers' comments:

Reviewer's Responses to Questions

**Comments to the Author**

1. Is the manuscript technically sound, and do the data support the conclusions?

The manuscript must describe a technically sound piece of scientific research with data that supports the conclusions. Experiments must have been conducted rigorously, with appropriate controls, replication, and sample sizes. The conclusions must be drawn appropriately based on the data presented.

Reviewer #1: Yes

Reviewer #2: Partly

**********

2. Has the statistical analysis been performed appropriately and rigorously?

Reviewer #1: Yes

Reviewer #2: No

**********

3. Have the authors made all data underlying the findings in their manuscript fully available?

The PLOS Data policy requires authors to make all data underlying the findings described in their manuscript fully available without restriction, with rare exception (please refer to the Data Availability Statement in the manuscript PDF file). The data should be provided as part of the manuscript or its supporting information, or deposited to a public repository. For example, in addition to summary statistics, the data points behind means, medians and variance measures should be available. If there are restrictions on publicly sharing data—e.g. participant privacy or use of data from a third party—those must be specified.

Reviewer #1: Yes

Reviewer #2: No

**********

4. Is the manuscript presented in an intelligible fashion and written in standard English?

PLOS ONE does not copyedit accepted manuscripts, so the language in submitted articles must be clear, correct, and unambiguous. Any typographical or grammatical errors should be corrected at revision, so please note any specific errors here.

Reviewer #1: Yes

Reviewer #2: No

**********

5. Review Comments to the Author

Please use the space provided to explain your answers to the questions above. You may also include additional comments for the author, including concerns about dual publication, research ethics, or publication ethics. (Please upload your review as an attachment if it exceeds 20,000 characters)

Reviewer #1: General comment

1. As this review aims to identify facilitators and barriers to interprofessional learning between nurses and physiotherapists in clinical hospital practice, I recommend structuring the findings accordingly. This structure would improve clarity, readability, and alignment with the review objectives.

2. Please provide reference for the statement provided in the results and discussion.

3. I recommend professional English language editing to enhance the clarity, coherence, and readability of the manuscript.

Abstract

- The title is not specific to older adults and could be revised to better reflect the study scope. Consider shortening the Background section of the abstract and including key methodological details, such as the review period and the number of databases searched, to improve clarity and completeness.

- You may consider to include the future direction of your study in the abstract.

Introduction

- The Introduction is rather lengthy. Consider condensing the background information and focusing on the key rationale, research gap, and objectives of the review.

- Rather than providing extensive background information, the authors should critically highlight the existing knowledge gap, the fragmented nature of the evidence, and the rationale for undertaking a scoping review.

Methods

- Line 99-100: The manuscript follows the Arksey and O’Malley framework; however, the subheadings are not aligned with its methodological stages. Consider revising the subheadings accordingly. Please include the PRISMA checklist in the supplementary.

- Line 105 – 108: You may consider to include the details of PCC mnemonic that used in this study to improve reader’s understanding.

- Line 111 – 113: Please include the reason of database selection and study duration.

- Line 128 – 130: The term “somatic adult hospital context” may not be familiar to an international audience. Consider replacing it with “adult hospital settings involving patients with physical health conditions” for greater clarity. You may consider to include the age range of this population.

- Line 130 – 131: The inclusion criteria are not clearly described and should specify based on the PCC mnemonic. The authors should also clearly state the type of evidence sources (quantitative/ qualitative/ mixed methods studies). I would recommend explicitly defining interprofessional learning (IPL) because some studies may use terms such as interprofessional education (IPE), workplace-based learning or interprofessional training.

- Line 131 – 134: The exclusion criteria should also be clearly described including study design, settings, populations, publication.

- Please clarify whether a quality assessment was undertaken. If not, provide a justification, as quality appraisal is not always required in scoping reviews but should be explicitly addressed.

Results

- I would suggest that the first results subheading focus solely on the characteristics of the included studies. The findings related to facilitators and barriers should be presented under separate subheadings to improve clarity and organization of the results.

- Study selection: Please specify whether studies from other methods (grey literature) in the search strategy. If excluded, provide a rationale for this decision

- Line 187: Please specify the age range and gender of the participants included in the review.

- Line 237: The term “digital team meetings” requires further clarification. Please describe the format of these meetings such as videoconferencing, virtual case discussions or online interprofessional learning session.

Discussion

- Line 360: The authors should consider discussing the potential limitations associated with restricting the search to specific languages and databases. These methodological choices may have excluded relevant evidence and influenced the overall scope and completeness of the review.

Reviewer #2: Comments

I would like to commend the authors for choosing a highly relevant and timely topic. This scoping review addresses a critical gap in health services literature by mapping the facilitators of and barriers to interprofessional learning specifically between nurses and physiotherapists in clinical hospital settings. The manuscript exhibits notable strengths, including a solid methodological foundation built on established scoping review frameworks, strict adherence to PRISMA-ScR guidelines, and proactive prospective protocol registration. The thematic synthesis of organizational conditions, team climates, and structural barriers is logically organized, providing valuable, actionable insights for clinical educators and hospital administrators alike. The manuscript holds clear merit and aligns well with the rigorous scope of PLOS ONE.

Despite these clear strengths, the manuscript requires a few essential revisions before it can be considered suitable for final publication. These recommendations primarily focus on correcting critical journal policy compliance issues, elevating methodological transparency, adding explicit data metrics with illustrative examples in the results, and deepening the overall analytical rigor of the discussion section. To assist the authors in maximizing the scholarly impact and readability of their research, I have outlined the essential revisions below.

1. Methodology and Data Support

•Quality Assessment: The manuscript does not address whether the included studies underwent quality appraisal. While quality assessment is not required for scoping reviews, acknowledging this limitation would enhance transparency.

You can add the following statement: "Consistent with scoping review methodology and the Arksey and O'Malley framework, formal quality assessment of the included studies was not performed. While this is a recognized feature of scoping reviews, readers should interpret the findings with consideration of the varying methodological quality of the primary studies."

•Enhance Transparency: Detail the screening consensus process, clarify if inter-rater reliability metrics (e.g., Cohen's kappa) were calculated, and justify omitting formal quality appraisal and statistical testing given the scoping review design.

•Risk of bias:

The review process involved independent screening by two authors, which is commendable. However, the manuscript could benefit from clearer description of:

How disagreements were resolved during title/abstract screening

Whether inter-rater reliability was calculated

The specific roles of each author in data extraction verification

•Strengthen Evidence and Metrics: Provide concrete examples from primary studies and state the exact study counts ($n$) contributing to each theme in both the Abstract and Results sections.

•Deepen Discussion: The Discussion currently tends to restate findings rather than analyze them in broader context. The connection to Vygotsky and Wenger is appropriate but could be more thoroughly developed. Move beyond descriptive summary by fully integrating Vygotsky’s and Wenger’s frameworks to explain findings, translating them into actionable recommendations for practice, education, and health policy. Briefly compare findings with other scoping reviews or systematic reviews on IPL in other contexts (e.g., primary care, community settings) to highlight what is unique about the hospital clinical practice context.

•The Results section presents findings through aggregated themes (facilitators: 3 themes; barriers: 3 themes) with multiple references per theme. While this is appropriate for scoping reviews, it lacks specific illustrative examples that would help readers understand how these factors manifest in practice.

•Expand the implications section to provide concrete recommendations:

For hospital administrators: specific strategies for creating shared physical spaces, scheduling regular interprofessional meetings, and leadership training.

For educators: how to better prepare students for interprofessional practice and support clinical supervisors.

For policymakers: implications for national guidelines and accreditation requirements.

2. Policy and Data Availability

•Correct Data Statement: Revise the Data Availability Statement to reflect that all underlying data are within Tables 1 and 2, removing inaccurate references to non-existent supporting files.

"All relevant data are within the manuscript and its Supporting Information files."

The authors claim data is in "Supporting Information files", but no such files were actually submitted with the manuscript.

It can be replaced by "All data generated or analyzed during this study are included in this published article (Table 1 and Table 2). No additional Supporting Information files are associated with this manuscript."

•Submit Missing Visuals: Upload the referenced PRISMA flowchart (Figure 1) as a separate, high-resolution image file instead of using a text placeholder.

•Include Mandated Declarations: Add an explicit Ethics Statement to the Methods section clarifying that ethical approval was not required for this secondary review of published literature.

You can add the following statement: "As this study constitutes a scoping review of previously published literature and did not involve human participants, animal subjects, or primary data collection, ethical approval was not required."

•Optimize Documentation: Consider moving full, database-specific search strings and raw extraction quotes into a dedicated Supporting Information file for complete replicability.

3. Language, Formatting, and Presentation

•Several language errors and awkward constructions throughout the manuscript. Inconsistent use of "interprofessional" vs. "inter-professional" (with hyphen) throughout the manuscript. Standardize it.

••Reformat Tables: Table 1 is dense and may be difficult for readers to navigate due to the amount of text in the "Facilitators" and "Barriers" columns. You can split it to tables.

•Verify Citations: Some references do not fully conform to PLOS ONE's reference formatting requirements.

Ref 1: Missing publisher location details

Ref 5: Incomplete journal information

Ref 6, 10: Inconsistent presentation of journal names

Format all references to strictly comply with PLOS ONE’s Vancouver style, ensuring full journal names, correct volume/page numbers, and missing DOIs.

Comments according to section:

Abstract: The abstract is well-structured and follows the standard format (Background, Method, Results, Conclusion).

Suggestions for improvement:

Background: Consider adding:

•One sentence on the importance of IPL for patient outcomes based on existing evidence.

Method: Add brief mention of:

•The databases searched

•Number of studies included (already included in Results)

Results: The results section could be slightly more quantitative:

"The database search yielded 1,973 articles, of which 11 were included. Facilitators included factors supporting socialization (n=X studies), positive team climate (n=X), and organizational conditions (n=X). Barriers included limited structural prerequisites (n=X), restricted interactions (n=X), and non-conducive workplace culture (n=X)."

Conclusion: Currently strong. Consider adding a sentence on the implications for policy or practice.

Introduction: The introduction effectively establishes the rationale for the study, provides relevant background on interprofessional collaboration, and justifies the focus on nurses and physiotherapists.

1.Add a paragraph on the prevalence of hospitalization among older adults and the consequences of functional decline to strengthen the clinical relevance.

2.Briefly acknowledge the existing evidence on barriers and facilitators to interprofessional collaboration more broadly to show this review builds on existing knowledge.

Methods: Strong methodological foundation.

1.Search strategy details: The search string is provided in text, but a table with the full search strategy for each database would be helpful for replicability. Consider adding as Supporting Information.

2.Gray literature: The manuscript does not explain why gray literature was excluded. Adding a brief justification would be helpful.

3.Scoping review vs. systematic review: Briefly justify why a scoping review is appropriate (e.g., to map the available evidence rather than answer a focused clinical question).

Results: Results are presented clearly with appropriate use of tables.

1.Add a brief description of the thematic analysis process in the Methods section (who developed the themes, how consensus was reached).

2.Provide numbers of studies contributing to each theme (quantitative summary) in addition to the references.

Discussion: Discussion connects findings to theory and practice but could be strengthened.

1.Compare findings with previous reviews: Briefly discuss how this review's findings align with or differ from previous reviews on interprofessional education and collaboration.

2.Add a subsection on "Implications for Research":

Need for studies specifically examining nurse-physiotherapist interaction

Need for longitudinal studies examining sustainability of IPL

Need for intervention studies testing specific IPL strategies

3.Add a subsection on "Implications for Education":

How can curricula be adapted to better prepare students for IPL?

What role should clinical supervisors play?

How can university-hospital partnerships be strengthened?

4.Add a subsection on "Implications for Practice":

Concrete recommendations for hospital administrators and ward managers

Specific strategies for overcoming identified barriers

Role of technology in facilitating IPL

Conclusion: Conclusion is appropriate and summarizes key findings.

Suggested Statement: "Hospital organizations and educational institutions must prioritize the development of sustainable IPL structures that transcend individual professionals and persist through staff turnover. Without such commitment, the potential for IPL to improve patient outcomes for older adults will remain unrealized."

**********

6. PLOS authors have the option to publish the peer review history of their article (what does this mean?). If published, this will include your full peer review and any attached files.

If you choose “no”, your identity will remain anonymous but your review may still be made public.

**Do you want your identity to be public for this peer review?**  For information about this choice, including consent withdrawal, please see our Privacy Policy.

Reviewer #1: No

Reviewer #2: No

**********

[NOTE: If reviewer comments were submitted as an attachment file, they will be attached to this email and accessible via the submission site. Please log into your account, locate the manuscript record, and check for the action link "View Attachments". If this link does not appear, there are no attachment files.]

To ensure your figures meet our technical requirements, please review our figure guidelines: https://journals.plos.org/plosone/s/figures

You may also use PLOS’s free figure tool, NAAS, to help you prepare publication quality figures: https://journals.plos.org/plosone/s/figures#loc-tools-for-figure-preparation.

NAAS will assess whether your figures meet our technical requirements by comparing each figure against our figure specifications.

Attachment Submitted filename: Manuscript Review - Facilitator and Barriers of IPL.docx

# Author comment: Author response to Decision Letter 1

**DOI:** 10.1371/journal.pone.0355722.r002

7 Jul 2026

General comments

- Please replace Figure 1 with the appropriate PRISMA diagram from the link: https://www.prisma-statement.org/prisma-2020-flow-diagram

Please find the appropriate PRISMA diagram for your study, download the Word document, and fill it out. Please state in your Methods section that you have followed this guideline and provide a reference citation and cite the completed checklist as a supplementary file.

Thank you for noticing this. We have now updated the Flowchart to the correct form according to PRISMA and added a statement in the Method section that we followed that guideline (see, p.6, lines 102-103 and Fig 1)

- Clearly highlight the inclusion and exclusion criteria of the included studies and the included population.

Thank you for this valuable comment. We agree and have revised the manuscript to provide a clearer description of the inclusion and exclusion criteria. Please see, pp.7-8, lines 139-146.

- Identify what “The population, concept, and context (PCC) mnemonic” is and how it was applied in the study.

We agree that the description was unclear. We have now revised the manuscript to provide a clearer explanation of how the PCC mnemonic was applied. Please see p.6, lines 114-117.

Reviewer 1

General comments

1. As this review aims to identify facilitators and barriers to interprofessional learning between nurses and physiotherapists in clinical hospital practice, I recommend structuring the findings accordingly. This structure would improve clarity, readability, and alignment with the review objectives.

Thank you for your valuable suggestions regarding the structure of the findings. In accordance with your comments, we have clarified the structure of the Results section (pp.9-14) to improve the clarity and readability of the manuscript. We have also revised Table 2 accordingly to ensure consistency throughout the manuscript.

2. Please provide reference for the statement provided in the results and discussion.

Thank you for noting this. We identified that references were missing for a few statements in the Results section. These have now been added, and the manuscript has been revised accordingly (p.11, line 209 and p.12, lines 239 and 241).

3. I recommend professional English language editing to enhance the clarity, coherence, and readability of the manuscript.

Thank you for your attention to this matter. As English is not the authors’ first language, the manuscript underwent professional language editing prior to submission. We have now carefully reviewed the manuscript once more and corrected the linguistic issues identified. We hope that the revised version is now clearer and linguistically improved.

Abstract

- The title is not specific to older adults and could be revised to better reflect the study scope.

Thank you for bringing this to our attention. As it was unclear which older population the study focused on, and since our methods did not restrict the population beyond adults (18 years and older), we have removed the specific focus on older adults and instead refer to adult in-hospital care throughout the manuscript. Consequently, we have not revised the title as suggested.

- Consider shortening the Background section of the abstract and including key methodological details, such as the review period and the number of databases searched, to improve clarity and completeness.

Thank you for this suggestion. Following your recommendation, we have shortened the Introduction section of the abstract to clarify the study rationale and expanded the methods section to improve the description of how the study was conducted, see the Abstract

- You may consider to include the future direction of your study in the abstract.

We agree that the future directions were not sufficiently clear. We have now included a statement in the concluding section of the abstract outlining future directions and the potential implications of the study. Please see the Abstract.

Introduction

- The Introduction is rather lengthy. Consider condensing the background information and focusing on the key rationale, research gap, and objectives of the review.

Rather than providing extensive background information, the authors should critically highlight the existing knowledge gap, the fragmented nature of the evidence, and the rationale for undertaking a scoping review.

Thank you for this valuable comment. Upon revisiting the Introduction, we agreed that it was unnecessarily long and did not sufficiently emphasize the knowledge gap and the rationale for conducting a scoping review. The Introduction has therefore been substantially shortened and refocused. Text that did not directly contribute to the study rationale was removed to better highlight the knowledge gap and the need for this review. Please see the revised manuscript (pp. 4–5).

Methods

- Line 99-100: The manuscript follows the Arksey and O’Malley framework; however, the subheadings are not aligned with its methodological stages. Consider revising the subheadings accordingly. Please include the PRISMA checklist in the supplementary.

Thank you for drawing our attention to this issue. We have now revised all method section subheadings to align with the Arksey and O’Malley framework. During this revision, we also recognized that Step 5 (Collating, Summarizing, and Reporting the Results) was previously insufficiently described. We therefore expanded this section to provide a clearer account of the analytical process and the reporting of the findings (see the manuscript, pp.6-9). We have also updated and included the PRISMA checklist as Supporting information (S1).

- Line 105 – 108: You may consider to include the details of PCC mnemonic that used in this study to improve reader’s understanding.

Thank you. As mentioned above, we have now revised the manuscript to provide a clearer explanation of how the PCC mnemonic was applied. Please see p.6, lines 114-117.

- Line 111 – 113: Please include the reason of database selection and study duration.

Thank you for this valuable comment. In response, we have clarified the rationale for the selection of databases and the chosen time frame in the Methods section of the manuscript (p. 8, lines 161–172).

- Line 128 – 130: The term “somatic adult hospital context” may not be familiar to an international audience. Consider replacing it with “adult hospital settings involving patients with physical health conditions” for greater clarity. You may consider to include the age range of this population.

Thank you for drawing our attention to this issue. We have now revised the description of the study setting to “adult hospital settings” or hospital-based clinical practice throughout the manuscript, including the abstract.

We considered a detailed explanation of somatic care unnecessary after explicitly adding psychiatric settings to the exclusion criteria (p.8, line 143). Furthermore, we removed the specific focus on older adults because it was not supported by our eligibility criteria, and added the age limit 18 years and older (p.6, line 117)

- Line 130 – 131: The inclusion criteria are not clearly described and should specify based on the PCC mnemonic. The authors should also clearly state the type of evidence sources (quantitative/ qualitative/ mixed methods studies). I would recommend explicitly defining interprofessional learning (IPL) because some studies may use terms such as interprofessional education (IPE), workplace-based learning or interprofessional training.

Thank you for this comment. We have revised the eligibility criteria to more explicitly reflect the PCC framework, clarifying the population (nurses and physiotherapists), concept (facilitators and barriers to IPL), and context (adult hospital settings). We have also specified that empirical studies only were eligible for inclusion, regardless of methodological approach. Furthermore, IPL is defined in the Introduction section (p.4, lines 62-64) and is now more clearly reflected in the search strategy. As the review specifically focused on workplace-based IPL rather than educational interventions, IPE-related terms were not included in the search strategy (pp.7-8, line 138-145).

- Line 131 – 134: The exclusion criteria should also be clearly described including study design, settings, populations, publication.

Thank you for this comment. We agree and have revised the exclusion criteria to more clearly specify which studies were excluded (pp.7-8, line 140-145).

- Please clarify whether a quality assessment was undertaken. If not, provide a justification, as quality appraisal is not always required in scoping reviews but should be explicitly addressed.

Thank you for this comment. We acknowledge the importance of study quality. However, in line with current scoping review methodology, a formal quality appraisal of included studies was not undertaken. To support scientific rigor, we restricted the inclusion criteria to peer-reviewed empirical studies. This rationale has now been clarified in the Methods section of the manuscript (p.8, lines 143-145).

Results

- I would suggest that the first results subheading focus solely on the characteristics of the included studies. The findings related to facilitators and barriers should be presented under separate subheadings to improve clarity and organization of the results.

Thank you for this comment. The text previously included in the Results section has been moved to the Methods section (Selecting the studies). We agree that the structure of the Results section needed to be clarified. We have therefore reorganized the section under the main headings Characteristics of the Included Studies, Facilitators of IPL in clinical practice, and Barriers to IPL in clinical practice, each with relevant subheadings. We believe this revision improves the clarity and organization of the findings (see, pp9-14).

- Study selection: Please specify whether studies from other methods (grey literature) in the search strategy. If excluded, provide a rationale for this decision

Thank you for this comment. It has now been clarified that grey literature was excluded because it had not undergone peer review, which was considered important for ensuring the quality of the included evidence. This has been specified in the manuscript (p. 8, lines 144–145).

- Line 187: Please specify the age range and gender of the participants included in the review.

Thank you for this observation. As the review included only healthcare professionals, all participants were adults of working age. Since Table 1 already contains extensive information and may be considered relatively dense, we chose not to further emphasize participants’ age and gender characteristics in the individual studies.

- Line 237: The term “digital team meetings” requires further clarification. Please describe the format of these meetings such as videoconferencing, virtual case discussions or online interprofessional learning session.

Thank you for noting this. We have now clarified in the manuscript that the digital team meetings referred to videoconferencing meetings (p.12, line 246)

Discussion

- Line 360: The authors should consider discussing the potential limitations associated with restricting the search to specific languages and databases. These methodological choices may have excluded relevant evidence and influenced the overall scope and completeness of the review.

Thank you for highlighting this important issue. The limitations related to the restriction to English-language publications and the selection of databases have now been acknowledged and clarified in the Limitations section of the manuscript (p.18, lines 377-390)

Reviewer 2

I would like to commend the authors for choosing a highly relevant and timely topic. This scoping review addresses a critical gap in health services literature by mapping the facilitators of and barriers to interprofessional learning specifically between nurses and physiotherapists in clinical hospital settings. The manuscript exhibits notable strengths, including a solid methodological foundation built on established scoping review frameworks, strict adherence to PRISMA-ScR guidelines, and proactive prospective protocol registration.

The thematic synthesis of organizational conditions, team climates, and structural barriers is logically organized, providing valuable, actionable insights for clinical educators and hospital administrators alike. The manuscript holds clear merit and aligns well with the rigorous scope of PLOS ONE.

Despite these clear strengths, the manuscript requires a few essential revisions before it can be considered suitable for final publication. These recommendations primarily focus on correcting critical journal policy compliance issues, elevating methodological transparency, adding explicit data metrics with illustrative examples in the results, and deepening the overall analytical rigor of the discussion section. To assist the authors in maximizing the scholarly impact and readability of their research, I have outlined the essential revisions below.

1. Methodology and Data Support

- Quality Assessment: The manuscript does not address whether the included studies underwent quality appraisal. While quality assessment is not required for scoping reviews, acknowledging this limitation would enhance transparency.

You can add the following statement: "Consistent with scoping review methodology and the Arksey and O'Malley framework, formal quality assessment of the included studies was not performed. While this is a recognized feature of scoping reviews, readers should interpret the findings with consideration of the varying methodological quality of the primary studies."

Thank you for noting this important point. We have revised the manuscript accordingly. Specifically, we clarified how we addressed the quality of the included studies (p.8 lines 143-145). We have also incorporated the proposed text into the Limitations section of the Discussion to acknowledge the absence of a formal quality assessment and its implications for the interpretation of the findings (p.18, lines 380-384).

Enhance Transparency:

- Detail the screening consensus process, clarify if inter-rater reliability metrics (e.g., Cohen's kappa) were calculated, and justify omitting formal quality appraisal and statistical testing given the scoping review design.

Thank you for this comment. We have clarified the screening and consensus process in the Methods section. Title- and abstract screening was conducted independently by two authors based on predefined inclusion and exclusion criteria, followed by full-text assessment. Any uncertainties were discussed with the third author until consensus was reached. Given the clear eligibility criteria and the narrative nature of the scoping review methodology, no inter-rater reliability statistics were calculated (pp. 7-8).

- We have also clarified that inclusion was restricted to peer-reviewed empirical studies. Consistent with scoping review methodology, no formal quality appraisal was conducted. To address the implications of this approach, as mentioned above, we have expanded the Limitations section and discussed how the absence of formal quality assessment may affect interpretation of the findings (p.18, lines 380-384).

Risk of bias:

- The review process involved independent screening by two authors, which is commendable. However, the manuscript could benefit from clearer description of:

How disagreements were resolved during title/abstract screening

Whether inter-rater reliability was calculated

Thank you for this comment. These issues raised have been addressed in our response above.

The specific roles of each author in data extraction verification

Thank you for this comment. The data charting process is now clarified under the heading “Charting the data”. Briefly, all data were charted by the first author and subsequently reviewed and verified by the two co-authors to ensure consistency and accuracy in the extracted data (pp. 8-9, lines 163-168).

Strengthen Evidence and Metrics:

- Provide concrete examples from primary studies and state the exact study counts ($n$) contributing to each theme in both the Abstract and Results sections.

Thank you for

Attachment Submitted filename: Response to reviewers.docx

# Peer review: Decision Letter 1

**DOI:** 10.1371/journal.pone.0355722.r003

**Authors:** Suad J Ghaben

26 Jul 2026

Facilitators of and barriers to interprofessional learning between nurses and physiotherapists in hospital-based clinical practice: A scoping review

PONE-D-25-59222R1

Dear Dr. **Åhlund,**

We’re pleased to inform you that your manuscript has been judged scientifically suitable for publication and will be formally accepted for publication once it meets all outstanding technical requirements.

Within one week, you’ll receive an e-mail detailing the required amendments. When these have been addressed, you’ll receive a formal acceptance letter and your manuscript will be scheduled for publication.

An invoice will be generated when your article is formally accepted. Please note, if your institution has a publishing partnership with PLOS and your article meets the relevant criteria, all or part of your publication costs will be covered. Please make sure your user information is up-to-date by logging into Editorial Manager at Editorial Manager® and clicking the ‘Update My Information' link at the top of the page. For questions related to billing, please contact billing support.

If your institution or institutions have a press office, please notify them about your upcoming paper to help maximize its impact. If they’ll be preparing press materials, please inform our press team as soon as possible -- no later than 48 hours after receiving the formal acceptance. Your manuscript will remain under strict press embargo until 2 pm Eastern Time on the date of publication. For more information, please contact onepress@plos.org.

Kind regards,

Suad J Ghaben, PhD

Academic Editor

PLOS One

Additional Editor Comments (optional):

Reviewers' comments:

Reviewer's Responses to Questions

**Comments to the Author**

1. If the authors have adequately addressed your comments raised in a previous round of review and you feel that this manuscript is now acceptable for publication, you may indicate that here to bypass the “Comments to the Author” section, enter your conflict of interest statement in the “Confidential to Editor” section, and submit your "Accept" recommendation.

Reviewer #1: All comments have been addressed

Reviewer #2: All comments have been addressed

**********

2. Is the manuscript technically sound, and do the data support the conclusions?

The manuscript must describe a technically sound piece of scientific research with data that supports the conclusions. Experiments must have been conducted rigorously, with appropriate controls, replication, and sample sizes. The conclusions must be drawn appropriately based on the data presented.

Reviewer #1: Yes

Reviewer #2: Yes

**********

3. Has the statistical analysis been performed appropriately and rigorously?

Reviewer #1: Yes

Reviewer #2: Yes

**********

4. Have the authors made all data underlying the findings in their manuscript fully available?

The PLOS Data policy requires authors to make all data underlying the findings described in their manuscript fully available without restriction, with rare exception (please refer to the Data Availability Statement in the manuscript PDF file). The data should be provided as part of the manuscript or its supporting information, or deposited to a public repository. For example, in addition to summary statistics, the data points behind means, medians and variance measures should be available. If there are restrictions on publicly sharing data—e.g. participant privacy or use of data from a third party—those must be specified.

Reviewer #1: Yes

Reviewer #2: Yes

**********

5. Is the manuscript presented in an intelligible fashion and written in standard English?

PLOS ONE does not copyedit accepted manuscripts, so the language in submitted articles must be clear, correct, and unambiguous. Any typographical or grammatical errors should be corrected at revision, so please note any specific errors here.

Reviewer #1: Yes

Reviewer #2: Yes

**********

6. Review Comments to the Author

Please use the space provided to explain your answers to the questions above. You may also include additional comments for the author, including concerns about dual publication, research ethics, or publication ethics. (Please upload your review as an attachment if it exceeds 20,000 characters)

Reviewer #1: Thank you for your feedback and the effort you have put into revising your work. I am pleased to see that you have carefully addressed all the comments and suggestions provided. All the best in your future endeavour.

Reviewer #2: I like to thank authors for their commitment and addressing the reviewer comments and wishing success to them

**********

7. PLOS authors have the option to publish the peer review history of their article (what does this mean?). If published, this will include your full peer review and any attached files.

If you choose “no”, your identity will remain anonymous but your review may still be made public.

**Do you want your identity to be public for this peer review?**  For information about this choice, including consent withdrawal, please see our Privacy Policy.

Reviewer #1: **Yes:**  Nurul Hidayah Md Fadzil

Reviewer #2: No

**********

# Editor report: Acceptance letter

**DOI:** 10.1371/journal.pone.0355722.r004

**Authors:** Suad J Ghaben

PONE-D-25-59222R1

PLOS One

Dear Dr. Åhlund,

I'm pleased to inform you that your manuscript has been deemed suitable for publication in PLOS One. Congratulations! Your manuscript is now being handed over to our production team.

At this stage, our production department will prepare your paper for publication. This includes ensuring the following:

* All references, tables, and figures are properly cited

* All relevant supporting information is included in the manuscript submission,

* There are no issues that prevent the paper from being properly typeset

You will receive further instructions from the production team, including instructions on how to review your proof when it is ready. Please keep in mind that we are working through a large volume of accepted articles, so please give us a few days to review your paper and let you know the next and final steps.

Lastly, if your institution or institutions have a press office, please let them know about your upcoming paper now to help maximize its impact. If they'll be preparing press materials, please inform our press team within the next 48 hours. Your manuscript will remain under strict press embargo until 2 pm Eastern Time on the date of publication. For more information, please contact onepress@plos.org.

You will receive an invoice from PLOS for your publication fee after your manuscript has reached the completed accept phase. If you receive an email requesting payment before acceptance or for any other service, this may be a phishing scheme. Learn how to identify phishing emails and protect your accounts at https://explore.plos.org/phishing.

If we can help with anything else, please email us at customercare@plos.org.

Thank you for submitting your work to PLOS ONE and supporting open access.

Kind regards,

PLOS ONE Editorial Office Staff

on behalf of

Dr. Suad J Ghaben

Academic Editor

PLOS One
